# Nonequilibrium Acceleration and Time Forecasting of Cluster-Mediated Self-Assembly

**DOI:** 10.1021/acs.jctc.5c01252

**Published:** 2025-11-22

**Authors:** Roy Furman, Michael Faran, Gili Bisker

**Affiliations:** † School of Electrical Engineering, Faculty of Engineering, 26745 Tel Aviv University, Tel Aviv 69978, Israel; ‡ School of Biomedical Engineering, Faculty of Engineering, Tel Aviv University, Tel Aviv 69978, Israel; § The Center for Physics and Chemistry of Living Systems, Tel Aviv University, Tel Aviv 6997801, Israel; ∥ The Center for Nanoscience and Nanotechnology, Tel Aviv University, Tel Aviv 6997801, Israel; ⊥ The Center for Light-Matter Interaction, Tel Aviv University, Tel Aviv 6997801, Israel; # The Center for Computational Molecular and Materials Science, Tel Aviv University, Tel Aviv 6997801, Israel

## Abstract

Nonequilibrium driving accelerates self-assembly by breaking the trade-off between thermodynamic stability and kinetic accessibility. While this principle has inspired a variety of theoretical and computational approaches, its effectiveness and predictability within physically realistic simulation frameworks remain to be systematically explored. Here, we investigate its impact using the Virtual-Move Monte Carlo (VMMC) method, a widely adopted approach for simulating collective particle dynamics during self-assembly. We investigate when such acceleration is both effective and predictable for three models, namely, VMMC with directed specific interactions, VMMC with undirected specific interactions, and an undirected single-particle Monte Carlo (SPMC), serving as a benchmark. Across all cases, nonequilibrium driving significantly reduces the time to first assembly, underscoring its robustness as a strategy for improving assembly efficiency. We further assess the Stochastic Landscape Method (SLM) as a forecasting tool for these models, and find its predictive power depends strongly on the nature of the interactions. Specifically, while SPMC and VMMC with undirected interaction show similar predictability, VMMC systems with directed interactions are more predictive than undirected dynamics. Analysis of simulation energy trajectories reveals the physical basis of these differences and delineates the conditions under which predictive tools like SLM are most effective. Our results highlight nonequilibrium driving as a powerful strategy for improving complex self-assembly outcomes and identify directed binding as a key principle for enhancing predictability.

## Graphical abstract

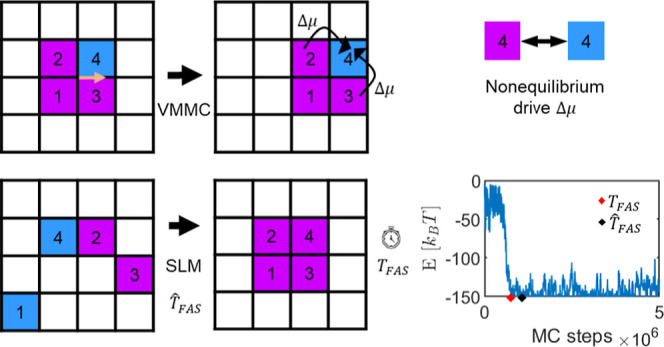

## Introduction

Self-assembly is a fundamental and pervasive phenomenon observed across biological, chemical, and synthetic systems,^1–6^ wherein individual components organize into well-defined structures without external guidance. This process underpins numerous natural systems, including protein folding,^7^ viral capsid formation,^8^ and the self-repair of cellular membranes.^9^ Likewise, self-assembly plays a crucial role in synthetic domains such as nanostructure fabrication,^10–13^ metamaterials,^14^ and advanced biomimetic platforms.^15^

Accurate simulations of self-assembly dynamics are essential for deepening our understanding and advancing applications in materials science,^16–18^ nanotechnology,^19–21^ and biomedical applications.^22–24^ A commonly used technique to simulate self-assembly is the Single-Particle Monte Carlo (SPMC),^25–28^ where particles’ locations and states are updated independently through local moves to explore the system’s configuration space. SPMC is computationally efficient, and effectively captures certain dynamical aspects. To include the realistic collective dynamics of oligomers^29^ and to sample essential intermediate states^30^ along cluster-mediated self-assembly pathways, where intermediate structures form first and then further assemble into larger, more complex architectures,^8,31–33^ Brownian dynamics (BD)^34–36^ and molecular dynamics (MD)^37–42^ are usually simulated to capture correlated motion.

Simulated correlated motion results in particles moving in a concerted fashion, yielding cluster dynamics. Several works have addressed this dynamical aspect in Monte Carlo simulations, capturing various features of realistic cluster behavior,^43–49^ attempting to imitate BD and MD success in capturing realistic cluster dynamics. To overcome the trade-off between physical realism and efficiency, the Virtual Move Monte Carlo (VMMC) algorithm was introduced by Whitelam and Geissler.^29,50^ VMMC enables the collective movement of strongly interacting particle clusters, facilitating the efficient sampling of physically realistic dynamics with a continuous-time interpretation of the MC steps. By capturing this realism, different kinetic observables are observed when compared to SPMC. Over the past decade, it has become a commonly employed method for simulating self-assembly.^51–55^

In self-assembly simulations, particle orientation critically affects both natural assembly and scenarios with external manipulation.^56–60^ Naturally, some systems adopt an undirected orientation interaction (UDI) between particle pairs, where the interaction between counterparts is not affected by their relative orientation angle.^61,62^ In contrast, directed interactions (DI) introduce orientation specificity, such that only a particular relative orientation angle between neighboring particles yields a strong interaction, while all other orientations are weaker or noninteracting. DI can be achieved through anisotropic particle design or external fields, and has been shown to alter assembly pathways and kinetics.^63–65^ Comparing natural and directed self-assembly systems reveals how particle orientation influences efficiency, structural predictability, and the ability to achieve targeted outcomes.^64,66–68^ Self-assembly can occur under equilibrium^69,70^ and nonequilibrium conditions.^71^ In the living cell, nonequilibrium effects have been shown to decrease assembly times^72^ and improve assembly stability.^73,74^ As a result, the study of self-assembly has increasingly turned to find nonequilibrium driving mechanisms to control and accelerate self-assembly,^5,34,75–82^ drawing inspiration from biological processes such as cytoskeletal remodeling^83^ and microtubule assembly.^84^ In particular, self-healing drives,^27,85^ periodic^79^ or feedback-based perturbations^82^ that mimic nature’s ability to correct structural errors, have been shown to enhance robustness and reduce assembly times in simulation frameworks. Incorporating such nonequilibrium driving in simulations has been shown to accelerate assembly and improve structural fidelity.^27,85,86^

To rationally design and optimize such nonequilibrium protocols, it is essential to predict the dynamics of assembly. A particularly relevant observable is the time to first assembly,^85,87^ i.e., the first temporal occurrence of the target structure, which serves as a proxy for kinetics and efficiency. To this end, the Stochastic Landscape Method (SLM) was recently developed,^27,82,88,89^ offering a framework to forecast assembly times based on trend change detection in simulation observables. While its predictive utility has been demonstrated in Single-Particle and Kinetic Monte Carlo simulations,^82^ its applicability to more physically realistic models involving collective particle dynamics, such as those captured by VMMC, remains unexplored.

In this work, we extend the SLM to analyze cluster-mediated nonequilibrium self-assembly under both SPMC and VMMC dynamics. We first examine how the time to first assembly depends on interaction energy in systems with undirected interactions, simulated using both SPMC and VMMC, under equilibrium and self-healing drive conditions. We then apply the SLM to each of these driven systems, including the VMMC model with directed interactions, to evaluate its predictive power for the first assembly time across different dynamical regimes. Finally, we investigate the physical origins of differences in predictability among the models by quantifying variability in energy trajectories and comparing these variability-based metrics to the predictive accuracy of the SLM. This analysis provides insight into the conditions under which self-assembly is not only accelerated but also forecastable. Our results provide insight into the importance of engineering directional interactions to minimize yield variability in biological and synthetic self-assembly, and of incorporating collective dynamics to better reflect realistic physical behavior in simulations. Looking ahead, the SLM can be utilized as a general framework for evaluating the predictability of driven systems in continuous space and time, offering a bridge between stochastic modeling and the rational design of functional, biologically inspired self-assembly materials.

## Methods

### Self Assembly Model

We simulate an attractive lattice gas in 2D, as depicted in Figure 1A. Our model consists of *N* distinguishable particles that can self-assemble into *M* target structures corresponding to predefined spatial arrangement of the building blocks that are encoded in the system’s memory via the interparticle interactions.^85^ Each particle is identified by a labeled number *i* = 1, ..., *N* and an internal state *s*_*i*_ = 1, ..., *M* corresponding to each target. The *M* targets are randomized at the beginning of each simulation, and are denoted as *S*_*m*_, where *m* = 1, ..., *M*. Each target has a unique internal state for all the particles, and thus each *s*_*i*_ state corresponds to a specific target *S*_*m*_.

**Figure 1:**
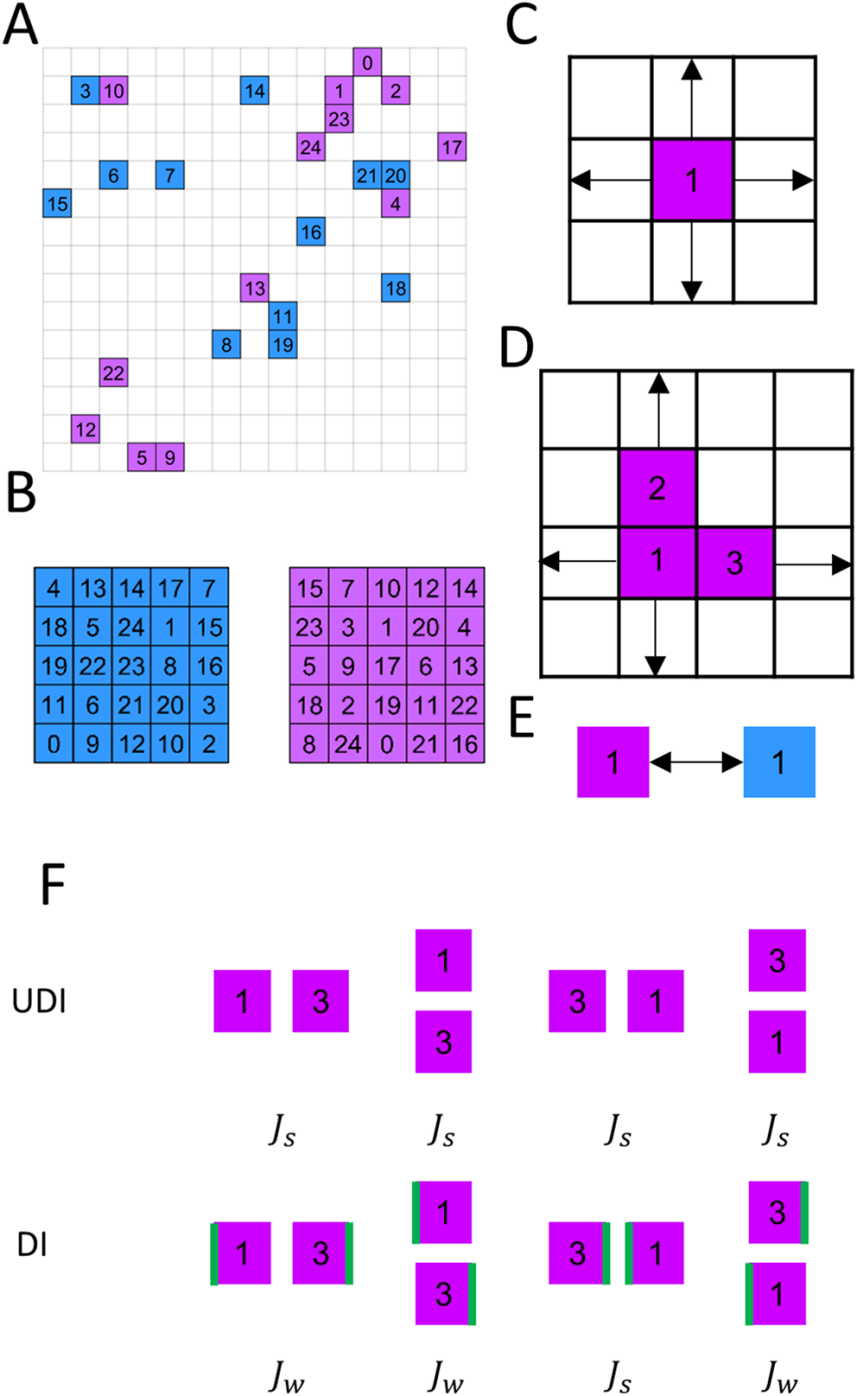
Model illustration. (A) Representative snapshot of the system on a 15 × 15 lattice containing *N* = 25 distinguishable particles, each assigned one of *M* = 2 internal states, represented by two distinct colors. Particles are uniquely labeled from 0 to 24. (B) Two target configurations, one for each internal state, define the system’s global energy minima, with each target specifying a unique arrangement of particle identities. (C) At each simulation step, a particle or a cluster (D) may undergo a physical move, accepted based on the Metropolis criterion. (E) Subsequently, a particle may undergo a state change, also according to Metropolis acceptance rule based on the associated energy change. (F) For UDI configuration, nearest neighbor particle pairs in a certain target exhibit interaction strength of *J*_s_ for all possible orientations. In contrast, for DI configuration, only one orientation exhibits interaction strength of *J*_s_ while the others are of *J*_w_.

The *N* particles are distributed on an *L* × *L* square lattice, where each site can either be occupied by a single particle or remain vacant. Particles can move across empty sites and switch between internal states while interacting with their neighboring particles. The simulation starts from random initial conditions for the particles’ locations and states.

In the self-assembly process, a target structure refers to a specific spatial arrangement of particles in which all particles share the same internal state (see Figure 1B). The simulations incorporate either directed interactions (DI) or undirected interactions (UDI). In the UDI case, the target structure is defined to include all rotated and mirrored variants of a given configuration, reflecting the orientation-invariant nature of the interactions (Figure 2). In contrast, for DI, only a single, uniquely oriented configuration qualifies as the target, consistent with the angular specificity imposed by directed binding.

**Figure 2:**
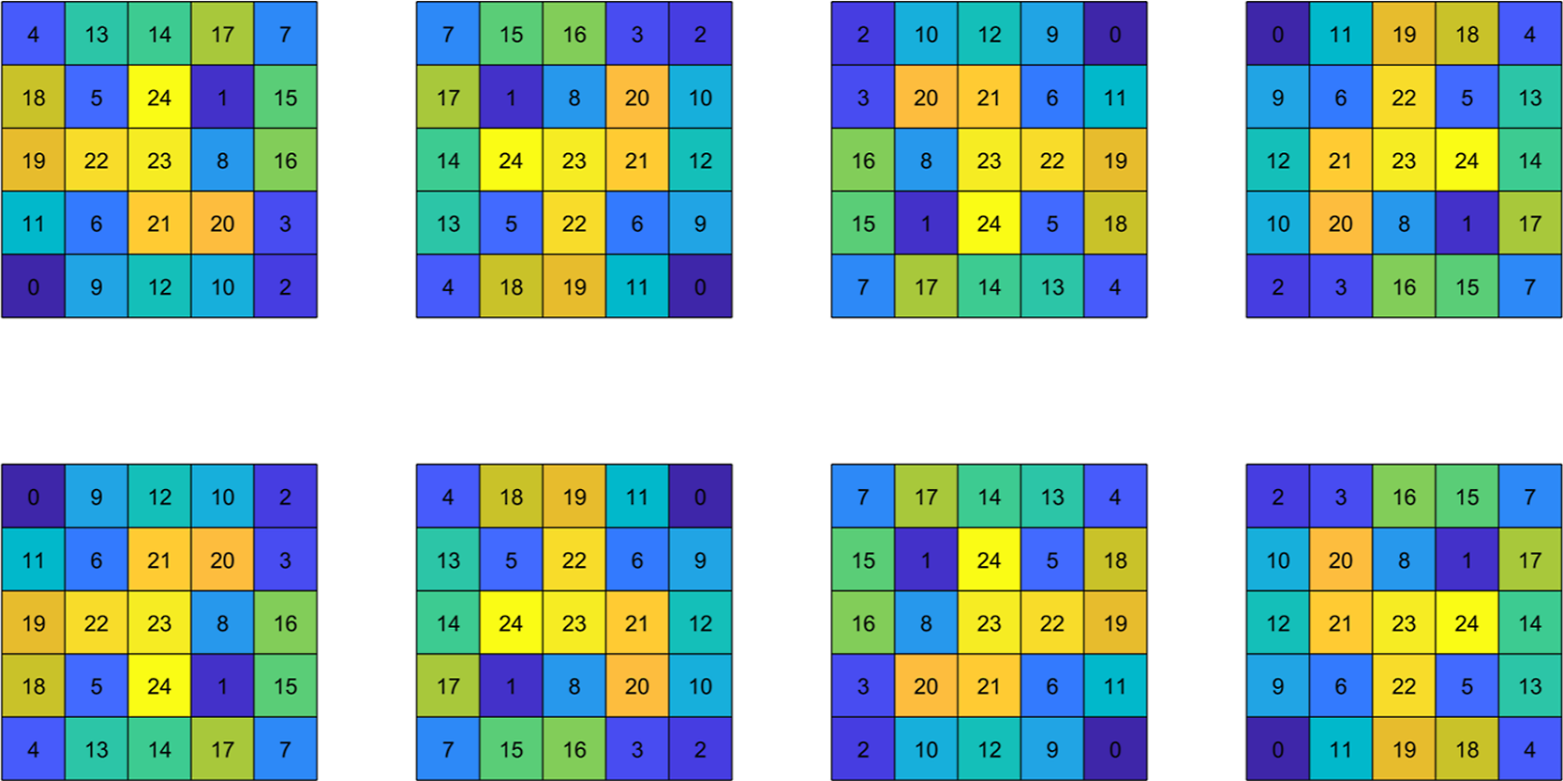
All possible orientations of a single target assembly of the undirected model. For the directed interaction configuration, only one orientation is accepted as the assembled target. Here, the colors aid in visualizing distinguished particles, and do not indicate different internal states.

When two particles *i* and *j* occupy adjacent lattice sites, they interact via a bond with energy *J*(*s*_*i*_, *s*_*j*_), whose magnitude depends on their internal states. A particle pair (*i*, *j*) is considered a neighboring pair, denoted as (*i*, *j*) ∈ *S*_*m*_, if they are nearest neighbors (n.n.) in at least one of the stored target structures for the UDI interaction. For the DI interaction, a particle pair (*i*, *j*) is considered a neighboring pair if they are n.n. and share the exact relative positioning as in the target structure (see Figure 1F).

If *i* and *j* are a neighboring pair in target *S*_*m*_, and both have internal state *s*_*i*_ = *s*_*j*_ = *m*, they experience a strong attraction of strength *J*_s_. Similarly, if they are a neighboring pair in both targets *S*_*m*_ and *S*_*n*_, and each particle is in the corresponding internal state (i.e., *s*_*i*_ = *m*, *s*_*j*_ = *n*, with *m* ≠ *n*), they also experience the strong interaction *J*_s_. If they are a neighboring pair only in target *S*_*m*_, and only one particle has the matching internal state (e.g., *s*_*i*_ = *m*, *s*_*j*_ ≠ *m*), the interaction strength is set to (*J*_s_ + *J*_w_)/2, where *J*_w_ is the weak interaction strength. If neither particle is in the correct internal state for the target structure, for which they are neighbors, or if they are not a neighboring pair in any stored structure, the interaction is *J*_w_. Mathematically, the interaction energy between adjacent particles *i* and *j* is expressed as

1 $$J\left(s_{i},s_{j}\right)=\{\begin{array}{ll} J_{s}, & \text{if},\left(i,j\right)\in S_{m},\text{and},s_{i}=s_{j}=m\\ J_{s}, & \text{if},\left(i,j\right)\in S_{m}\cap S_{n},\text{and},s_{i}=m,s_{j}=n,,m\neq n\\ \frac{J_{s}+J_{w}}{2}, & \text{if},\left(i,j\right)\in S_{m},\text{and},\left(s_{i}=m\;\text{or}\;s_{j}=m\right),,s_{i}\neq s_{j}\\ J_{w}, & otherwise \end{array}$$

and the total energy of the system, *E*, is computed as

2 $$E=\sum_{j>i}^{N}J\left(s_{i},s_{j}\right)$$

The dynamics is then simulated by the Virtual Move Monte Carlo (VMMC) algorithm, with the option for UDI or DI interactions, or by the Single Particle Monte Carlo (SPMC) algorithm with UDI. Each iteration of the simulation consists of two substeps: a physical move and a state-switch. The first step selects a random particle and attempts to move it to a new adjacent site in a randomly chosen direction, namely, up, down, left, or right, with periodic boundary conditions. If the new site is already occupied, the move is rejected. Otherwise, if SPMC dynamics are considered as in Figure 1C, the move is accepted or rejected according to the Metropolis-Hastings condition^25^ with an energy difference argument following eqs 1 and 2. This condition respects detailed balance, and thus reflects equilibrium conditions.

If the Virtual-Move Monte Carlo (VMMC) algorithm is employed, the randomly selected particle, referred to as the seed particle, initiates a process that may result in a collective cluster move in the randomly chosen direction.^29^ First, one of the seed particle’s nearest neighbors is randomly selected. The seed particle then performs a virtual move, meaning its displacement is temporarily applied to a cloned simulation state. This allows for the computation of the postmove energy when the bond between the seed and its neighbor is hypothetically broken. The system is then immediately reverted to its original state, while the resulting energy change is stored in memory.

Based on the energy difference between the pre- and postmove configurations and a prefactor based on the tentative cluster size, a recruitment probability is calculated for the selected neighbor, and a Bernoulli trial is performed using this probability. If the trial succeeds, the neighbor is added to the tentative cluster move, consisting of two particles; otherwise, the bond is considered broken. Additionally, the reverse recruitment or bond-breaking probability, used later in the final acceptance step, is also computed. It is defined by the movement of the tentative cluster in the opposite direction.

The process continues with additional nearest neighbors. If the neighbor is not recruited, the algorithm proceeds to examine the remaining n.n. of the seed particle. If the neighbor is recruited, the recruitment test is iteratively applied to its n.n. along with the seed particle neighbors. For each neighbor, the same steps are followed: a virtual collective move of the tentative cluster is attempted, the energy difference is calculated, a Bernoulli trial is conducted, and both forward and reverse recruitment probabilities are recorded. This process continues iteratively until no further neighbors of particles within the cluster remain to be tested.

The accumulated result of bond-creation and rejection trials, manifested as a multiplication of virtual moves acceptance and rejection probabilities, is the generation probability of the proposed one MC step cluster move. Although the Bernoulli trials probabilities used for accepting or rejecting were part of the cluster generation process, they also participate in the final VMMC acceptance probability calculation.^29^ Thus, while constructing a cluster C by virtual linking, finalizing the particle recruitment process for a single MC step in VMMC, a trial move from microstate ζ to η is finally accepted with the following probability^29^

3 $$W_{acc}^{\left(\zeta \to \eta |C\right)}=\Theta \left(n_{c}-n_{C}\right)D\left(C\right)\min\left\{1,e^{-\left(E_{\eta }-E_{\zeta }\right)}\frac{\prod q_{ij}\left(\eta \to \zeta \right)}{\prod q_{ij}\left(\zeta \to \eta \right)}\frac{\prod \left(\eta \to \zeta \right)}{\prod p_{ij}\left(\zeta \to \eta \right)}\right\}$$

here, *E*_α_ is the system energy in microstate α, D(C)≤1 modulates the diffusivity of C (e.g., imposes $D\left(C\right)\propto n_{C}^{-0.5}$ , where $n_{C}$ is the cluster size, measured by the number of particles in the cluster) to capture physical cluster diffusion rates consistent with three-dimensional Stokes drag, similar to Holmes and Wyart.^55^ $\Theta \left(n_{c}-n_{C}\right)$ is the Heaviside step function that enforces a sampled cluster size cutoff *n*_c_, so all particles are accepted for motion with comparable frequency, as *n*_c_ drawn as the smallest integer larger than ξ^–1^, where ξ is a random number in (0,1]. The factors *p*_*ij*_ and *q*_*ij*_ ≡ 1 – *p*_*ij*_ are, respectively, the probabilities to form or not form links between particles *i* and *j* during the forward/backward virtual-move construction; products over *q*_*ij*_ correct for failed links internal/external to C , while products over *p*_*ij*_ correct for formed links. The link probability used during construction is

4 $$p_{ij}\left(\zeta \to \eta \right)=\Theta \left(n_{c}-n_{C}\right)J_{ij}^{\left(\zeta \right)}\;\max\left[0,1-e^{-\left[\varepsilon^{\prime }\left(i,j\right)-\varepsilon \left(i,j\right)\right]}\right]$$

where $J_{ij}^{\left(\zeta \right)}=1$ if *i* and *j* interact in ζ (else 0), ε­(*i*, *j*) is the pair energy of bond *ij* in ζ, and ε′(*i*, *j*) is the bond energy after a virtual move of *i* relative to *j*. Both ε­(*i*, *j*) and ε′(*i*, *j*) calculations follow eq 2, and are determined by the respective particle labels. Equations 3 and 4 ensure superdetailed balance^29^ and are physically consistent with single-particle diffusion rates per one MC step with regard to the simulation timesteps clock, and promote physically consistent collective moves when neighbors experience energy changes under the virtual displacement. An example of possible cluster moves is depicted in Figure 1D.

The second step randomly selects a different particle and assigns it a new internal state, as in Figure 1E, distinct from its current one. Under equilibrium conditions, the energy difference associated with this state change is computed, and the switch is accepted with a probability determined by the standard Metropolis criterion. We emphasize that this process is not recursive, as only a single particle can change its state at a time.

To explore how nonequilibrium perturbations influence self-assembly dynamics, we incorporate a self-healing drive that biases internal state changes based on local particle environments, mimicking biologically inspired error-correction mechanisms. Specifically, we introduce a driving parameter Δμ that modulates the likelihood of a particle changing its internal state according to that of its neighbors. If a selected particle has two or more n.n. sharing the same internal state, the probability of switching its state to match them increases by an exponential factor of Δμ. Conversely, if the particle’s current state is already shared by at least two of its neighbors, the probability of switching to a different state decreases by Δμ. For example, if a particle has four n.n. divided into two same-state pairs, the net driving force would be zero. The acceptance probability *q*_flip_, for an internal state switch with associated state energy change of Δ*E* under external driving is computed similarly to our previous works^27,85^

5 $$q_{flip}=\min\left\{1,e^{-\Delta E\pm \Delta \mu }\right\}$$

These modified dynamics violate detailed balance and, hence, are out of equilibrium as the Entropy Production Rate (EPR) was shown to increase when the drive mechanism was deployed.^85^ To align with the Metropolis-Hastings criterion, we define Δμ with a negative sign so that the exponential term increases with the absolute value of Δμ. This formulation promotes assembly by favoring configurations where neighboring particles share the same state.

We simulate T MC iterations containing the two steps of a physical move and state-switch, and update the simulation parameters accordingly after each iteration. We retain in memory the energy trajectories versus the MC iteration and sample them each *n*_MC_ steps, referred to as the binning factor. We refer to the sampled MC iterations as sweeps, meaning that the total number of sweeps is $T_{sweeps}=\frac{T}{n_{MC}}$ . Observing the sampled trajectories, a target is considered assembled for energy values of $2J_{s}\sqrt{N}\left(\sqrt{N}-1\right)$ where all particle pair interactions in a square of *N* particles (as in Figure 1B) attain the value of *J*_s_. The MC step at which the particles form one of the targets and the system reaches its global energy minimum is denoted as *T*_FAS_.

The simulation parameters used in this study are summarized in Table 1. The rationale for these choices follows our previous work^27^ and is further detailed in the Supporting Information. The default strong interaction energy, |*J*_s_|, was defined as the lowest value at which more than 50% of the SPMC simulation realizations successfully completed at least one assembly event within the allotted T MC steps.

**Table 1:** Default Simulation Parameters

parameter	notation	value
number of particles	*N*	25
grid size	*L*	15
number of states	*M*	2
strong interaction	*J*_s_	–4 [*k*_B_T]
weak interaction	*J*_w_	–1 [*k*_B_T]
drive	Δμ	[−0, −0.5, −1, −1.5, −2] [*k*_B_T]
binning factor	*n*_MC_	[5000, 500]
total MC steps	T	[5 × 10^7^, 5 × 10^6^]

Moreover, the binning factor was set to *n*_MC_ = 500 for DI simulations and *n*_MC_ = 5000 for UDI simulations, reflecting the faster assembly kinetics and more frequent structural changes observed in the DI configuration. T was set to 5 × 10^6^ for DI simulations, and 5 × 10^7^ otherwise. For additional information, please refer to the Supporting Information, Figures S1–S10.

### The Stochastic Landscape Method

The Stochastic Landscape Method (SLM) is a data-driven approach for forecasting nonequilibrium self-assembly times,^27^ rooted in transition state theory^90^ and is described below, summarizing our previous work. The SLM segments time-series trajectories of macroscopic 1D observables (e.g., total energy or collective variables^88^) using the Bayesian Estimator of Abrupt Change, Seasonality, and Trend (BEAST) algorithm, identifying temporal regions with distinct statistical properties.^91^ By extracting the mean, standard deviation, and trend of observables within these segments, referred to as “the stochastic coordinates”. Stochastic coordinates are extracted from an ensemble of observable trajectories corresponding to a given simulation configuration. Each trajectory is truncated at its respective *T*_FAS_ if an assembly event occurs, while trajectories without successful assembly are excluded from the analysis. The SLM constructs a predictive map based on the extracted data, referred to as the stochastic landscape. This stochastic landscape relates the system’s current segment state, defined by its stochastic coordinates, to the remaining time until self-assembly. Specifically, the remaining time to first assembly is defined as the difference between the MC step at which the first assembly event occurs and the onset time of the segment from which the prediction is made.

To evaluate predictive performance, the SLM is applied to new segmented trajectory data (the test set) from the same simulation configuration. Stochastic coordinates are extracted for each segment, allowing the model to estimate the remaining time to first assembly. These predictions are then compared to the actual measured values for each segment. As a baseline, the prediction accuracy is also evaluated against a naïve estimator that assigns the median *T*_FAS_ as the remaining time to assembly for all segments. As in our previous work,^82^ the SLM does not require any input parameters besides the trajectory data. It assumes the underlying BEAST algorithm involves linear trends and practically no limitations on the number of trend-change points or the minimum time between trend-change points.

In this work, we apply the SLM to predict the remaining time to first assembly in VMMC simulations, for both DI and UDI scenarios. To assess its performance, we compare it to SPMC-based prediction by evaluating the Pearson correlation coefficient, *R*, between the measured and SLM-estimated logarithm of the remaining time to first assembly across different simulation configurations. Beyond evaluating SLM accuracy, we also interpret *R* as a meaningful figure of merit for the system’s inherent predictability. To this end, we compare it with two alternative metrics that quantify the predictability of a specific observable: the standard deviation (STD) of *T*_FAS_, following Shukla’s approach,^92^ and the root-mean-square deviation (RMSD) from the mean energy trajectory.^93,94^ Both the STD and the RMSD are computationally efficient and robust methods for quantifying variability among trajectories, facilitating the clear interpretation of consistency and predictability in the self-assembly process across multiple stochastic simulations.

To compute the RMSD, we constructed the average energy trajectory, *E*_avg_(*t*), by calculating the mean energy value at each simulation time step (*t*) across the ensemble of simulation trajectories that eventually reach the target structure, and are used to build the stochastic landscape. At each time step, this average is computed using only those trajectories for which the first assembly event has not yet occurred. The RMSD for each individual energy trajectory, *E*(*t*), is then calculated by measuring its deviation from the average trajectory over the time interval up to its unique *T*_FAS_

6 $$RMSD=\sqrt{\frac{1}{T_{FAS}}\sum_{t=1}^{T_{FAS}}{\left(E\left(t\right)-E_{avg}\left(t\right)\right)}^{2}}$$

Although the RMSD and STD characterize different aspects of variability than the SLM correlation coefficient *R*, we hypothesize that these three metrics are meaningfully related. In particular, high variability in energy trajectories (reflected by RMSD) is expected to lead to increased dispersion in *T*_FAS_ values. In turn, greater variability in *T*_FAS_ implies less consistency in the remaining time to assembly across segments, which directly impacts the predictability captured by *R*. Thus, we anticipate that systems with more stable energy trajectories and narrower *T*_FAS_ distributions will exhibit higher SLM predictability.

For each simulation configuration, we calculate the STD of *T*_FAS_ and the RMSD of energy trajectories across the ensemble. Higher values of these metrics indicate greater variability and, consequently, lower predictability. To evaluate their relationship with SLM performance, we compare the SLM-derived *R* values against the inverse RMSD and inverse STD across all configurations. Pearson correlation coefficients are then computed to quantify the association between *R* and each of these inverse variability metrics. For clarity, we note that *R* here refers to the Pearson correlation coefficient obtained from the SLM’s prediction of the remaining time to first assembly. These *R* values are subsequently used as input for an additional correlation analysis to assess how well SLM predictability aligns with variability-based measures.

## Results and Discussion

To investigate how nonequilibrium driving and interaction specificity affect cluster-mediated self-assembly, we performed Monte Carlo simulations using two dynamical schemes: Single-Particle Monte Carlo (SPMC) and Virtual-Move Monte Carlo (VMMC), both implemented in a discrete-time, discrete-space framework. Each model was tested under both equilibrium and nonequilibrium (self-healing drive) conditions, and included either directed (DI) or undirected (UDI) particle interactions. For each configuration, we analyzed the time to first assembly, *T*_FAS_, across a broad range of interaction strengths and drive magnitudes.

We then applied the Stochastic Landscape Method (SLM) to evaluate its ability to predict the remaining time to assembly from energy trajectory data, and compared its predictive performance to standard variability-based metrics such as the standard deviation (STD) of *T*_FAS_ and the root-mean-square deviation (RMSD) of energy trajectories.

### Simulation Models Comparison

We begin by analyzing the assembly kinetics as a function of drive strength Δμ, comparing the performance of the VMMC and SPMC models under undirected interaction configurations. Figure 3 presents the median *T*_FAS_ values computed from 200 simulation realizations for each configuration. Following the work of Whitelam and Geissler,^29^ where aggregation processes were compared across different simulation models using a characteristic time scale *t*_d_, defined as the time for a monomer to diffuse a distance equal to its diameter, we adopt the same convention here. In our model, each monomer corresponds to a single lattice tile, so *t*_d_ is naturally equivalent to one Monte Carlo step for both models. Moreover, in line with the rigorous treatment of Sanz and Marenduzzo,^95^ the Monte Carlo step can be consistently taken as the time scale for both algorithms, since their formulation shows that, at the monomeric level, particles of equal size, diffusion coefficient, and acceptance probability of a single particle yield an equivalent mapping between Monte Carlo and physical time. In both SPMC and VMMC, the acceptance and rejection rates are calibrated with respect to the same fundamental time scale. Because single-particle events have identical durations in the two algorithms, this calibration ensures that the mapping from event frequencies to physical time is consistent. Consequently, simulation times in SPMC and VMMC are automatically synchronized relative to the base unit of single-particle motion. The results reveal the range of binding energies for which more than half of the realizations successfully assemble the target structure within the allotted simulation duration, capped by the total number of iterations, T . Therefore, the plateau at the top of each figure illustrates that more than half of the trajectories for that model did not reach assembly before T iterations.

**Figure 3:**
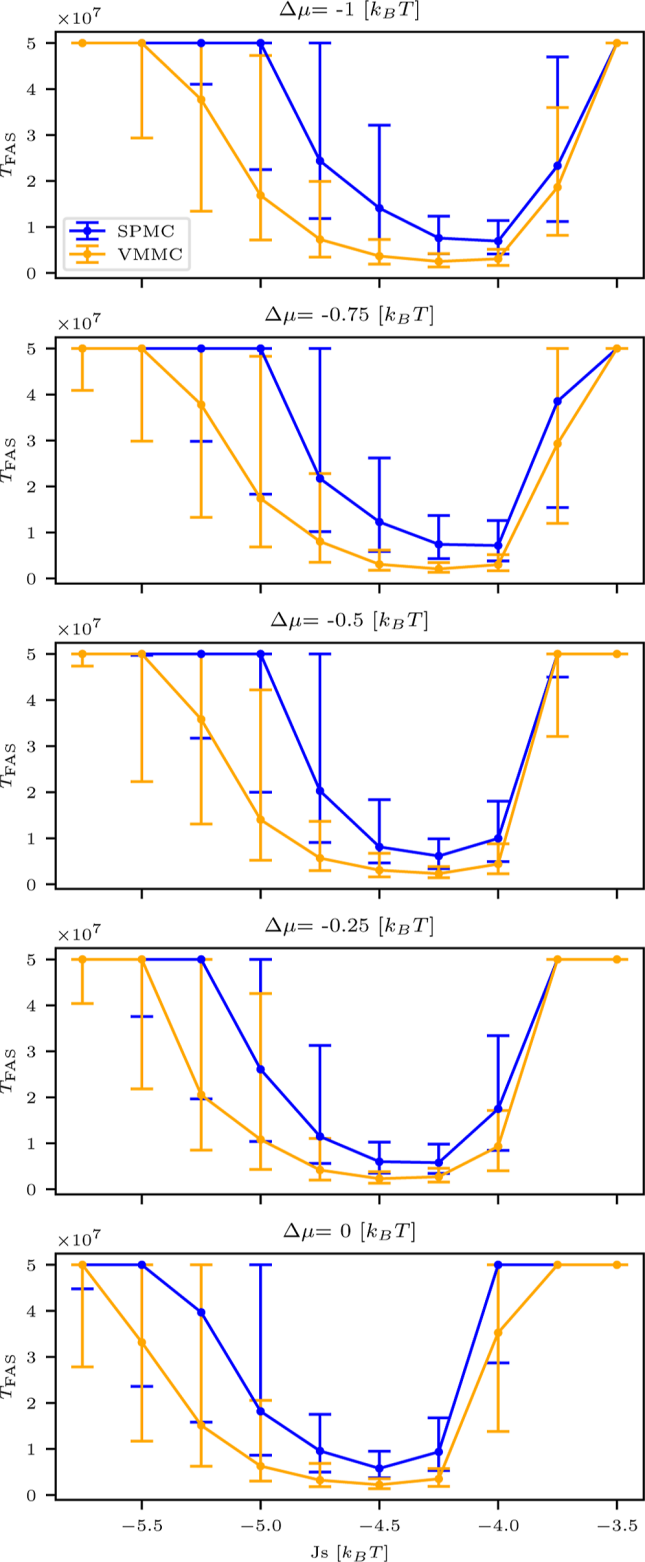
Median *T*_FAS_ as a function of the strong binding energy *J*_s_ for SPMC (blue) and VMMC (orange) UDI simulations, calculated for different drive values Δμ. The whiskers represent the first and third quartiles.

A key observation is that this assembly window systematically shifts toward lower absolute values of the binding energy |*J*_s_| as the magnitude of the drive |Δμ| increases for both dynamical schemes. This shift arises from the dual role of the nonequilibrium drive across different interaction regimes. At weak binding energies, nucleation seeds are unstable due to insufficient interaction strength. However, the drive enhances local alignment by favoring internal state agreement among neighboring particles, thereby stabilizing early assembly seeds. Once nucleation occurs, assembly proceeds efficiently through a self-correcting process in which incorrect, weakly bound configurations are naturally destabilized and replaced with correct ones.^82^ At stronger binding energies, however, increasing |Δμ| can become counterproductive. The drive may inadvertently stabilize incorrect or misaligned substructures, making them resistant to rearrangement. These kinetically trapped configurations impede proper growth and increase the time required to reach the target structure.^82^

The interplay of these two effects, namely, drive-assisted nucleation at low |*J*_s_| and drive-induced trapping at high |*J*_s_|, results in a shifted assembly window that depends on the magnitude of the applied drive. This behavior is consistently observed in both the SPMC and VMMC simulations, as shown in Figure 3, suggesting a general, algorithm-independent mechanism.

We further observe that the VMMC model facilitates faster assembly than the SPMC model, as indicated by its lower median *T*_FAS_ values across the full range of examined Δμ. This difference highlights the more efficient exploration of the configuration space enabled by the collective moves in the VMMC algorithm, which reduce kinetic trapping and accelerate progression toward the target structure. A more detailed discussion of this trend, including its physical implications and dependence on model parameters, is presented later in the article.

Continuing with the default binding energy *J*_s_ = −4 [*k*_B_ *T*] (see Table 1), we examine the distribution of *T*_FAS_ for both SPMC and VMMC under UDI configurations, as shown in Figure 4. The distributions were fitted using kernel density estimation (KDE) alongside a log–normal model, following the approach used in our previous work.^27^ To ensure consistency, the fits were truncated at the highest observed *T*_FAS_ value in each configuration, and no extrapolated values are shown. As seen in Figure 4 and consistent with prior results,^27^ increasing the drive strength improves the agreement between the empirical *T*_FAS_ distribution and the log–normal fit. Across all examined drive values, the KDE and log–normal models exhibit similar behavior, supporting the use of log–normal statistics to describe assembly times under nonequilibrium conditions. Intuitively, the observed log–normal fit of the first-assembly time distribution can be rationalized as follows: a subset of trajectories proceeds without kinetic stalls (unimpeded nucleation and growth), producing the short-time rise, whereas rare trap-escape events generate a long right tail.^96^ This behavior is more pronounced at larger |Δμ| in Figure 4. We hypothesize that increasing drive favors same-particle-state microstates and, consequently, same-state macrostates in the assembled structure, effectively reducing the accessible state space and rendering the dynamics more homogeneous. In this more homogeneous, low-detachment-probability regime, separated fast and slow nucleation time scales naturally emerge, consistent with previous homogeneous assembly analytic analysis.^96^ A model-specific derivation for our system is left for future work.

**Figure 4:**
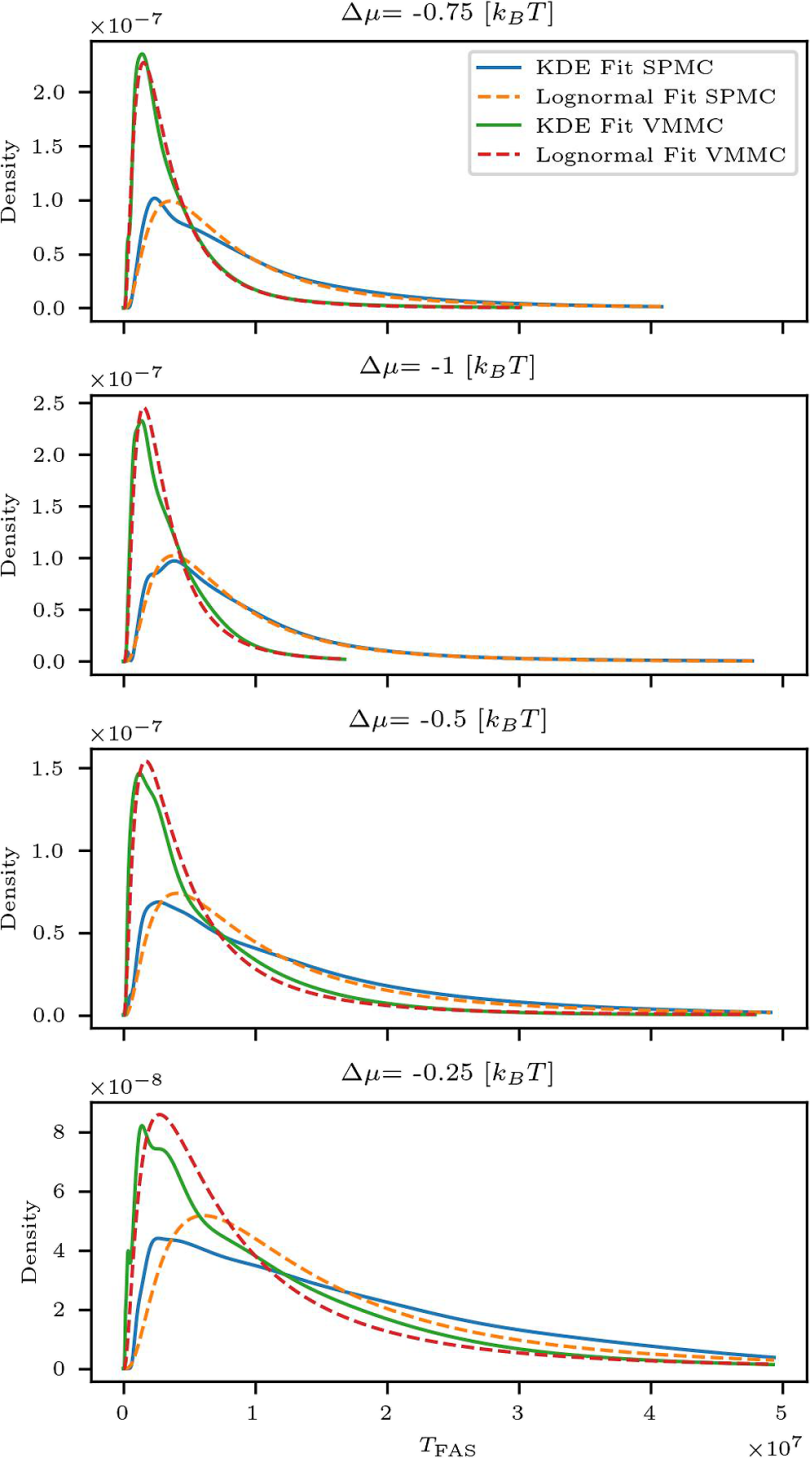
Comparison of the *T*_FAS_ distributions for SPMC and VMMC simulations under undirected interactions, shown for varying drive strengths Δμ at fixed binding energy *J*_s_ = −4 [*k*_B_ *T*]. Distributions are fitted using kernel density estimation (KDE) and log–normal models. The KDE for SPMC is shown as a solid blue line, and for VMMC as a solid green line. The corresponding log–normal fits are shown as dashed yellow for SPMC and dashed red for VMMC.

Figure 5 shows the median *T*_FAS_ values for both the SPMC and VMMC UDI configurations as a function of drive strength Δμ, at fixed binding energy *J*_s_ = −4 [*k*_B_ *T*]. In both models, *T*_FAS_ decreases monotonically with increasing |Δμ|, consistent with previous findings.^27^ This trend suggests that similar physical mechanisms underlie the influence of nonequilibrium driving in both dynamical schemes.

**Figure 5:**
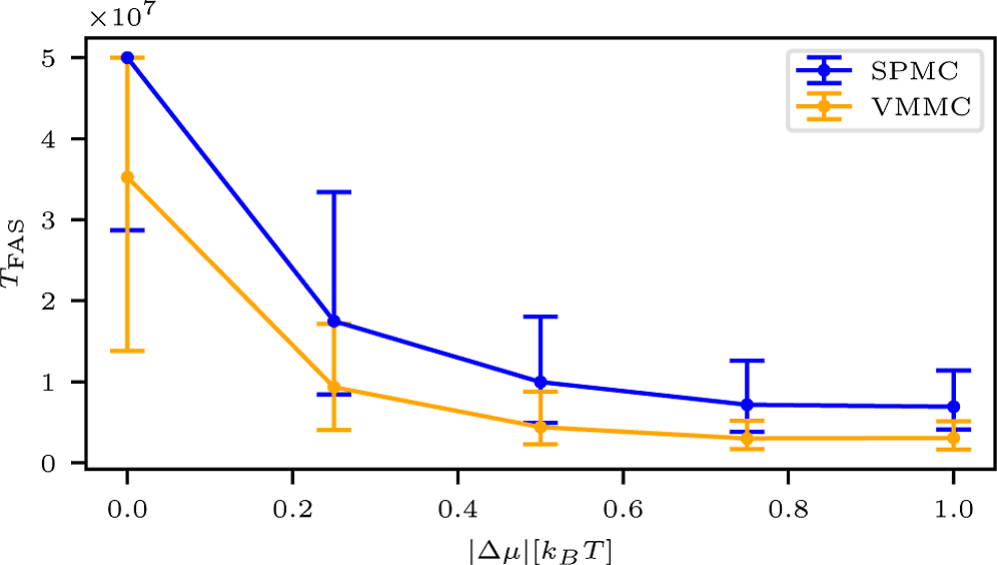
*T*_FAS_ as a function of the drive value for strong binding energy value *J*_s_ = −4 [*k*_B_ *T*], for SPMC (blue) and VMMC (orange). The whiskers represent the first and third quartiles.

Across all drive values, the VMMC model exhibits consistently shorter median *T*_FAS_ compared to SPMC. This behavior is also evident across other values of *J*_s_ (see Figure 3), underscoring the generally faster assembly kinetics achieved with VMMC. The enhanced efficiency of VMMC can be attributed to its cluster-based update strategy, which allows for collective particle motions and early rejection of energetically unfavorable moves. This includes the ability to avoid forming or preserving incorrect bonds during virtual move trials, whereas SPMC relies on random, sequential single-particle updates to resolve such misassemblies. As a result, VMMC promotes more effective error correction and accelerates the system’s progression toward the target structure.

To further compare these dynamics, Supporting Information Figures S11 and S12 present the median energy trajectory and the maximum cluster size, along with their 25% to 75% interquartile ranges, respectively, obtained from a set of 15 trajectories for each drive value. These figures highlight the faster and consistent growth observed in VMMC simulations compared to SPMC, illustrating the difference in assembly pathways of the two simulation types across different drive strengths. Figure S13 then compares the median *T*_FAS_ values as a function of the drive value for both VMMC UDI and DI configurations on a logarithmic scale. The figure highlights an order-of-magnitude difference in assembly times, with DI configurations assembling significantly faster. Despite the magnitude difference, both exhibit the same qualitative trend of reduced *T*_FAS_ with increasing drive strength. This suggests that directed interactions follow the same fundamental mechanism of drive-enhanced first stable assembly and error correction. Finally, Supporting Information Figures S14 and S15 compare VMMC–UDI and VMMC–DI by plotting the median and the 25–75% interquartile range (IQR) for both energy trajectories and maximum cluster size, using 15 trajectories per drive value. These results show that, beyond faster assembly, DI yields more consistent pathways than UDI. A detailed kinetic comparison, SPMC vs VMMC and DI vs UDI, with quantified uncertainty (Tables S2 and S3) is provided in the Supporting Information subsection on assembly pathways heterogeneity analysis.

### The SLM Predictability Comparison

We assess the predictive performance of the Stochastic Landscape Method across three simulation types, namely, SPMC with undirected interactions, VMMC with undirected interactions, and VMMC with directed interactions. For this analysis, we generate a simulation ensemble for each combination of simulation method and drive value listed in Table 1. Each simulation configuration, defined by a specific method–drive pair, consists of 800 independent realizations of the assembly dynamics. From each ensemble, 640 trajectories are randomly selected to construct the stochastic landscape, following the approach described in our prior work.^27^ We emphasize that in this work, the SLM method operates on the total-energy trajectory, motivated by transition state theory and our prior results.^27^ The framework is observable-agnostic to any kinetics–informative reaction coordinate. For some systems, where self-assembly is driven by entropy,^97^ choosing a different baseline observable may be more informative. In general, selecting the optimal one-dimensional geometrical descriptor or reaction coordinate for any system is not a trivial task, and existing frameworks^98,99^ can assist in this process. Thus, the SLM can be extended to incorporate alternative reaction coordinates that may provide improved estimators.

This procedure incorporates a bias correction procedure, repeated ten times: trajectory segments are randomly partitioned into training and cross-validation sets. A stochastic landscape constructed from the training set is used to predict first assembly times for the cross-validation set. For each split, a binned prediction error is computed, and the results are averaged across all repetitions to produce the final bias correction vector. The remaining 160 trajectories serve as a held-out test set, used to evaluate the SLM’s ability to predict the remaining time to first assembly based on the extracted stochastic coordinates.

The predictive performance of the SLM across different simulation methods for Δμ = −1 [*k*_B_ *T*] is illustrated in Figure 6 (top row), which presents scatter plots comparing the measured remaining time to first assembly in log scale (*Y*_test_) against the SLM predictions before and after bias correction, denoted as ${Ŷ}_{p}$ and ${Ŷ}_{BC}$ , respectively, for SPMC (Figure 6A) and VMMC (Figure 6B) undirected interaction models, and for VMMC with directed interactions (Figure 6C). To visualize the accuracy of the bias-corrected predictions, we apply logarithmic binning (bin width = 0.5) to ${Ŷ}_{BC}$ along the *x*-axis. For each bin, we generate a boxplot of the corresponding *Y*_test_ values. A linear fit is applied to the medians of these boxplots to show the overall trend of measured versus predicted values, as shown in Figure 6 (middle row).

**Figure 6:**
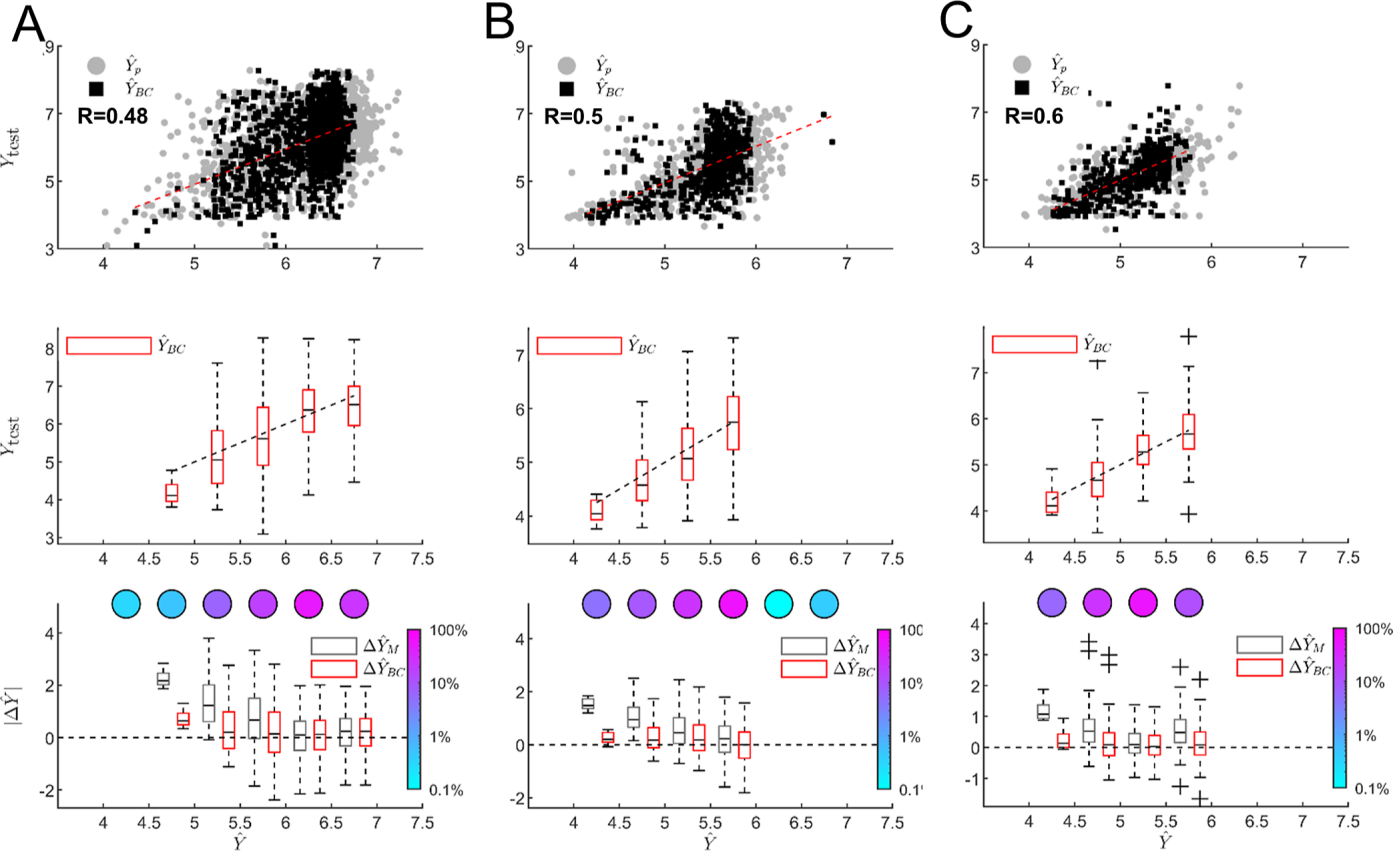
SLM prediction performance for (A) SPMC with undirected interactions (UDI), (B) VMMC with UDI, and (C) VMMC with directed interactions (DI), respectively, all with Δμ = −1 [*k*_B_ *T*] and *J*_s_ = −4 [*k*_B_ *T*]. Top row: Scatter plots comparing the actual measured values (log scale, *Y*_test_) with the predicted remaining time to first assembly (log scale, Ŷ ) for each segment. Gray dots represent SLM predictions before bias correction ( ${Ŷ}_{p}$ ), and black dots show bias-corrected predictions ( ${Ŷ}_{BC}$ ). Red lines indicate linear regression fits to the data. Middle row: Binned boxplots of the same predictions, using logarithmic bins of width 0.5 along the ${Ŷ}_{BC}$ axis. Each box represents the distribution of *Y*_test_ within a bin, with whiskers denoting the interquartile range and outliers shown as plus signs. The dashed diagonal line (*y* = *x*) indicates perfect agreement between predicted and actual values. Bottom row: Comparison of prediction errors for the bias-corrected SLM predictor and a naïve median-based predictor. Red boxplots represent the absolute error Δ*Y*_BC_ between ${Ŷ}_{BC}$ and *Y*_test_; black boxplots show the error Δ*Y*_*M*_ when using the global median of *T*_FAS_ as a constant predictor. The colorbars visualize the percentage of the total Ŷ samples contained within each bin on a logarithmic scale, and appear above each bin in correspondence.

To quantitatively assess prediction accuracy, we compare the error of the bias-corrected predictor ${Ŷ}_{BC}$ to that of a baseline predictor ${Ŷ}_{M}$ , which is taken as the log-median of *T*_FAS_ across all segments. The prediction error $\Delta {Ŷ}_{BC}$ is calculated for each test point as the absolute value of the difference between ${Ŷ}_{BC}$ and *Y*_test_. Similarly, $\Delta {Ŷ}_{M}$ is computed by replacing ${Ŷ}_{BC}$ with the constant median value ${Ŷ}_{M}$ for all points. These error values are aggregated within each logarithmic bin and displayed as boxplots in Figure 6 (bottom row), with a horizontal dashed line indicating zero error for reference. This comparison highlights the improvement offered by the SLM over a naïve median-based predictor across the range of predicted values.

Figure 6 (bottom row) shows that the SLM’s predictive accuracy varies with the segment’s temporal proximity to the final assembly event. For segments far from the target structure, corresponding to a high number of remaining Monte Carlo steps (rightmost boxplots), the SLM performs comparably to the baseline median predictor. However, as the system nears assembly completion, the SLM substantially outperforms the median-based prediction, demonstrating its enhanced effectiveness in forecasting when the system is closer to the target. This behavior is observed consistently across all three simulation types. When the system is far from the target, the number of accessible stochastic pathways increases, making accurate forecasting inherently more difficult regardless of the kinetic model.

The corresponding Pearson correlation coefficients, shown in the top row of Figure 6, further quantify these observations. Both the SPMC and VMMC simulations with undirected interactions yield comparable predictive performance with *R* ≈ 0.5 (Figure 6A,B). In contrast, the VMMC simulation with directed interactions achieves a higher correlation of *R* = 0.6 (Figure 6C), indicating improved alignment between predicted and actual assembly times. This suggests that interaction directionality contributes more significantly to predictability than the simulation method itself. We hypothesize that the moderate Pearson correlation coefficients primarily reflect the inherent stochastic nature of the system, which imposes fundamental limits on predictability,^100^ rather than any intrinsic limitations of the SLM approach.

To further explore the role of interaction directionality in predictability, we examine the SLM’s Pearson correlation coefficient *R* across additional simulation configurations. These results are summarized in Table 2, where *R*_VMMC_^DI^, *R*_VMMC_^UDI^, and *R*_SPMC_^UDI^ represent the predictive performance of the VMMC with directed interactions (DI), VMMC with undirected interactions (UDI), and SPMC with UDI, respectively. While *R*_VMMC_^UDI^ and *R*_SPMC_^UDI^ yield comparable values across the examined drive strengths, *R*_VMMC_^DI^ consistently exhibits higher correlation values for all tested conditions. This trend reinforces the conclusion that interaction directionality plays a more critical role in enhancing predictability than the choice of simulation algorithm.

**Table 2:** Pearson Correlation Coefficient (*R*) between the Predicted and Actual Remaining Time to First Assembly for the Different Simulation configurations and Across Driving Strengths

Δμ	*R*_VMMC_^DI^	*R*_VMMC_^UDI^	*R*_SPMC_^UDI^
0.25	0.72	0.36	0.46
0.5	0.74	0.49	0.43
0.75	0.59	0.5	0.46
1.0	0.6	0.5	0.48
1.25	0.62	0.52	0.42

The strong contribution of interaction directionality to predictability can be understood intuitively. In UDI simulations, particle pairs, and by extension, clusters, can form in any relative orientation (see Figure 2). This flexibility often leads to misaligned subclusters that, although strongly bonded, are incorrectly oriented with respect to the target structure. For full assembly to occur, such subclusters must disassemble and reorient, creating kinetic traps that increase variability in the assembly process. In contrast, DI simulations promote the formation of clusters with consistent relative orientations, reducing the likelihood of such misassemblies and enabling more direct and predictable pathways toward the target configuration.

Finally, we compare the SLM-derived predictability metric *R* with two alternative indicators of system predictability: the inverse of the root-mean-square deviation (RMSD) and the inverse of the standard deviation (STD) of *T*_FAS_, denoted σ­(*T*_FAS_). These comparisons are based on the simulation configurations presented in Table 2, and follow the methodology described in the Methods section. The resulting scatter plots are shown in Figure 7, along with linear regression lines for both cases. The Pearson correlation coefficient between *R* and the inverse RMSD was calculated to be 0.75, indicating a strong positive relationship between SLM predictability and the consistency of energy trajectories (Figure 7, top). Similarly, the correlation between *R* and the inverse of σ­(*T*_FAS_) is 0.71 (Figure 7, bottom), highlighting a comparable relationship between SLM predictability and the temporal variability of first assembly times. These correlations support our hypothesis that SLM moderates Pearson correlations stemming from the limited predictability of the system. These results suggest that RMSD and σ­(*T*_FAS_) serve as meaningful and complementary metrics to the SLM-derived *R*, collectively capturing different aspects of a system’s predictability.

**Figure 7:**
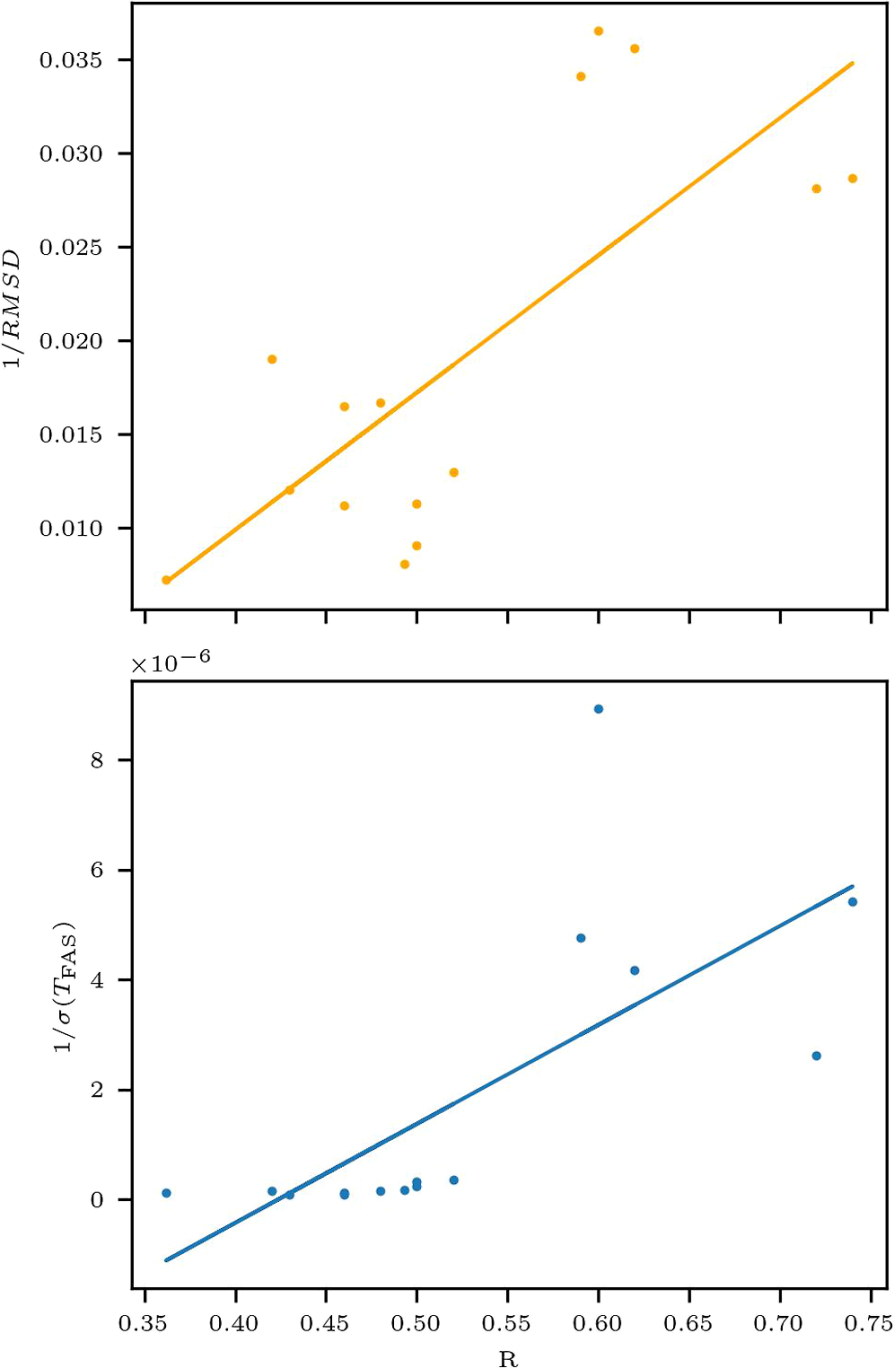
Comparison of predictability metrics across simulation configurations. Top panel: Scatter plot of the inverse RMSD (1/RMSD) versus the SLM-derived predictability metric (*R*), with a solid linear regression line indicating the trend. Bottom panel: Scatter plot of the inverse standard deviation of first assembly time (1/σ­(*T*_FAS_)) versus *R*, with a solid linear regression line.

We reiterate that the presented results are based on a lattice-based toy model, chosen for its computational efficiency and tractability, with the hypothesis that the fundamental physical principles governing assembly extend to continuous space and time for certain systems. In addition, we consider a single set of particles, i.e, one copy of the assembled structure and local environment, under the assumption of a globally well-mixed environment, reflected in periodic boundary conditions of the lattice.^101^ This framework, in which particles are uniformly distributed and interact with equal probability, intentionally abstracts away spatial heterogeneity to reveal the essential interplay between interaction strength, nonequilibrium driving, and assembly efficiency at a fundamental level. Moreover, we analyze a relatively small system to capture key physical insights, where implementations for larger systems and alternative models are left for future work.

## Conclusions

In this work, we investigated the effects of nonequilibrium driving and directed interactions on the efficiency and predictability of cluster-mediated self-assembly using Virtual Move Monte Carlo (VMMC) simulations, comparing them with Single-Particle Monte Carlo (SPMC) dynamics on a discrete-time lattice model. We systematically analyzed variations in first assembly times across simulation configurations and evaluated the ability of the Stochastic Landscape Method (SLM) to predict these times under nonequilibrium conditions. Additionally, we assessed SLM-based predictability against standard metrics such as RMSD and STD of *T*_FAS_. Consistent with prior work,^27,28,40,41,85,86,102–105^ our results confirm that nonequilibrium driving accelerates self-assembly at a specific binding energy range, whereas higher binding energies may impede assembly by stabilizing chimeric intermediates and promoting kinetic traps.

Among systems with undirected interactions, VMMC outperformed SPMC in achieving faster first assembly times. We attribute this advantage to two factors: (1) VMMC’s acceptance or rejection criteria reduce sampling of unwanted off-target states, which results from virtual moves as preceding steps preventing their occurrence, and (2) its collective particle moves enable broader exploration of favorable pathways. Introducing directed interactions into VMMC further improved assembly kinetics, likely by promoting cluster formation in near-target orientations and suppressing kinetic traps.

We next evaluated the SLM’s performance in predicting the remaining time to first assembly. Across all configurations tested, the SLM predictions showed strong correlations with measured values of assembly times. The SLM notably outperformed a median-based prediction baseline, especially in the later stages of the assembly process when the system is close to the target. These results highlight SLM’s potential as a data-assimilation tool for forecasting assembly progression under nonequilibrium conditions.

Importantly, SLM analysis revealed that interaction directionality has a stronger influence on predictability than the choice of simulation method. While SPMC and VMMC exhibited similar predictive performance under undirected interactions, directed interactions yielded more predictable assembly times. Although predicting nonequilibrium systems remains fundamentally challenging,^100,106^ we demonstrated that SLM-based predicted values correlate well with established variability-based metrics such as the RMSD and STD of *T*_FAS_, suggesting that the SLM not only serves as a forecasting tool, but also as a quantitative proxy for evaluating the predictability of dynamic assembly systems. Moreover, the SLM’s robust predictive power and physical interpretability suggest its potential as a framework for constructing emergent models of complex stochastic systems.

These findings open avenues for future work, including testing SLM-based control protocols,^82^ exploring nonreciprocal interactions,^28^ examining the SLM prediction performance for other one-dimensional observables than the energy, and integrating alternative predictive approaches^102,103^ to develop general design principles for out-of-equilibrium assembly. Additional work could extend this approach to other self-assembly systems, including continuous time and space models, explore alternative predictability metrics such as entropy production, and simulate larger, more realistic systems such as viral capsid assemblies.^8,107^

This work is part of ongoing efforts^55,104,105,108^ to bridge recent theoretical and simulation advances in self-assembly with realistic experiments, aiming to uncover meaningful design principles and emergent rules in both biological and synthetic contexts.

## Supplementary Material



## Data Availability

The data and software used to create this research are available upon request.

## Notes

The Supporting Information contains additional figures and analyses that complement the main results. These include a section explaining the choices of the simulation physical parameters, a detailed view of how the first assembly time (*T*_FAS_) varies between simulation types (SPMC vs VMMC), strong binding strengths (*J*_s_), and nonequilibrium driving levels (Δμ), highlighting trends and differences not included in the main text. Figure S1: Boxplots of *T*_FAS_ vs *J*_s_ for SPMC at Δμ = 0, Figure S2: Boxplots of *T*_FAS_ vs *J*_s_ for VMMC at Δμ = 0, Figure S3: Boxplots of *T*_FAS_ vs *J*_s_ for SPMC at Δμ = −0.25 [*k*_B_ *T*], Figure S4: Boxplots of *T*_FAS_ vs *J*_s_ for VMMC at Δμ = −0.25 [*k*_B_ *T*], Figure S5: Boxplots of *T*_FAS_ vs *J*_s_ for SPMC at Δμ = −0.5 [*k*_B_ *T*], Figure S6: Boxplots of *T*_FAS_ vs *J*_s_ for VMMC at Δμ = −0.5 [*k*_B_ *T*], Figure S7: Boxplots of *T*_FAS_ vs *J*_s_ for SPMC at Δμ = −0.75 [*k*_B_ *T*], Figure S8: Boxplots of *T*_FAS_ vs *J*_s_ for VMMC at Δμ = −0.75 [*k*_B_ *T*], Figure S9: Boxplots of *T*_FAS_ vs *J*_s_ for SPMC at Δμ = −1.0 [*k*_B_ *T*], Figure S10: Boxplots of *T*_FAS_ vs *J*_s_ for VMMC at Δμ = −1.0 [*k*_B_ *T*], Figure S11: Kinetic pathways difference of the energy, compared between SPMC and VMMC, Figure S12: Kinetic pathways difference of the Maximum cluster size, compared between SPMC and VMMC, Figure S13: Comparison of *T*_FAS_(Δμ) for DI vs UDI configurations at *J*_s_ = −4 [*k*_B_ *T*] Figure S14: Kinetic pathways difference of the energy, compared between DI and UDI, Figure S15: Kinetic pathways difference of the Maximum cluster size, compared between DI and UDI, Table S1: Average of the interquartile range values along the median energy trajectory for all simulation types, Table S2: Average of the interquartile range values along the median Maximum cluster size trajectory for all simulation types (PDF)
