# Supplementary material for: Nonequilibrium Acceleration and Time Forecasting of Cluster-Mediated Self-Assembly
Source: J Chem Theory Comput. 2025 Nov 22;21(23):11880–93. doi: 10.1021/acs.jctc.5c01252 (PMC12874364; doi:10.1021/acs.jctc.5c01252)
Supplement: Supplementary file 1 [file ct5c01252_si_001.pdf]

Supporting Information

Nonequilibrium Acceleration and Time Forecasting of Cluster-Mediated Self-Assembly

Roy Furman,† Michael Faran,‡ and Gili Bisker*,‡,¶,§,‖,⊥

†*School of Electrical Engineering, Faculty of Engineering, Tel Aviv University, Tel Aviv 69978, Israel*

‡*School of Biomedical Engineering, Faculty of Engineering, Tel Aviv University, Tel Aviv 69978, Israel*

¶*The Center for Physics and Chemistry of Living Systems, Tel Aviv University, Tel Aviv 6997801, Israel*

§*The Center for Nanoscience and Nanotechnology, Tel Aviv University, Tel Aviv 6997801, Israel*

‖*The Center for Light-Matter Interaction, Tel Aviv University, Tel Aviv 6997801, Israel*

⊥*The Center for Computational Molecular and Materials Science, Tel Aviv University, Tel Aviv 6997801, Israel*

E-mail: bisker@tauex.tau.ac.il

S1 Additional Model Analysis

S1.1 Directed Interactions Time to First Assembly Analysis

The target structure is defined as a 5×5 square lattice, corresponding to a total of $N = 25$ particles. Larger system sizes were not considered, as they would substantially increase computational demands and limit our ability to systematically explore a broad parameter space. To balance computational feasibility with sufficient assembly space, the grid size is set to $L \times L$ with $L = 15$. This allows the particles to assemble into the target structure within a reasonable simulation time. The total number of Monte Carlo steps, $\mathscr{T}$, was chosen based on the characteristic first assembly time, T_{FAS}, following the approach used in our previous work.[1] The weak interaction energy was fixed at $J_w = -1$ $[k_B T]$, ensuring that $|J_w| < |J_s|$ for all values of the strong interaction energy J_s used in this study. To investigate the effect of interaction strength on T_{FAS} under both equilibrium and nonequilibrium conditions, the strong interaction parameter J_s was varied over the range $[-5.75, -3.5]$ $[k_B T]$. The number of encoded targets was set to $M = 2$, which captures the key features of the internal state-switching dynamics without introducing unnecessary complexity or excessive computational cost.

We present below the box plots referenced in the main text, which quantify the dependence of T_{FAS} on J_s across different simulation conditions. Unless otherwise stated, all simulation parameters follow the defaults listed in Table 1 of the main text, with only the drive $\Delta\mu$ and strong interaction strength J_s varied. Each box plot represents the distribution of T_{FAS} values obtained from multiple simulation realizations. The boxes indicate the interquartile range (IQR), whiskers extend to data points within 1.5 times the IQR, and outliers beyond this range are shown as circles.

Figs. S1–S2 depict results under equilibrium conditions, while Figs. S3–S10 show results

for driven systems. Across all conditions, a consistent tradeoff emerges between kinetic accessibility and thermodynamic stability. At low $|J_s|$, weak binding interactions prevent the formation of stable assembly seeds, leading to long assembly times or failure to assemble. At intermediate $|J_s|$, assembly is most efficient, as interactions are strong enough to support growth while remaining weak enough to allow error correction, defining the so-called "assembly window" described in the main text. At high $|J_s|$, although the thermodynamic stability of the target structure, if formed, is enhanced due to strong binding, the system is prone to kinetic traps that stabilize incorrect intermediates. These misassembled structures hinder progression toward the target, again resulting in delayed or unsuccessful assembly.

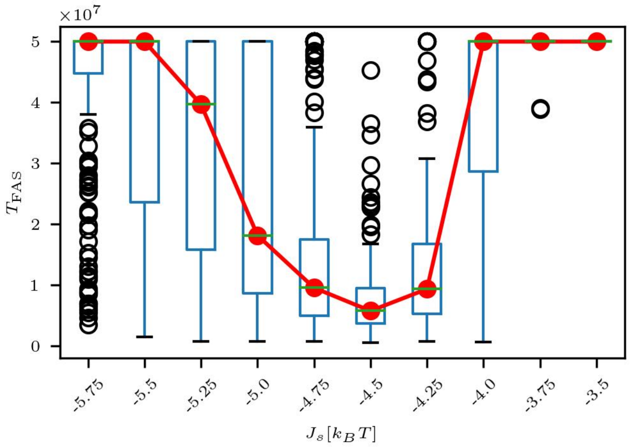

Figure S1: Boxplost of T_{FAS} across multiple J_s values for undirected SPMC simulation configuration with $\Delta\mu = 0$.

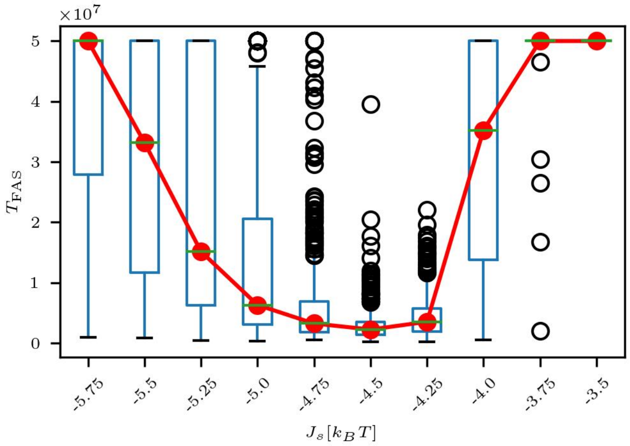

Figure S2: Boxplots of T_{FAS} across multiple J_s values for undirected VMMC simulation configuration with $\Delta\mu = 0$.

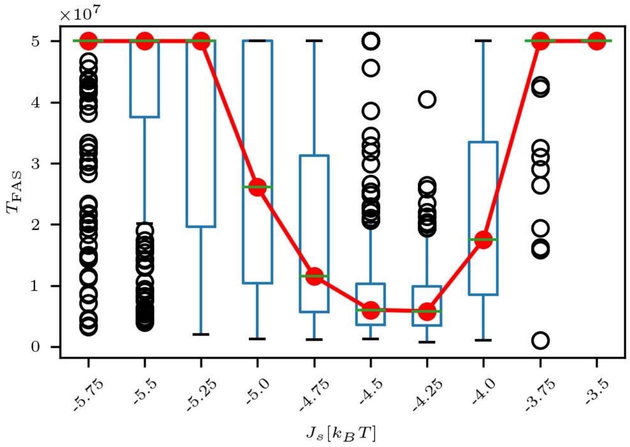

Figure S3: Boxplots of $T_{\rm FAS}$ across multiple J_s values for undirected SPMC simulation configuration with $\Delta\mu = -0.25$ $[k_BT]$.

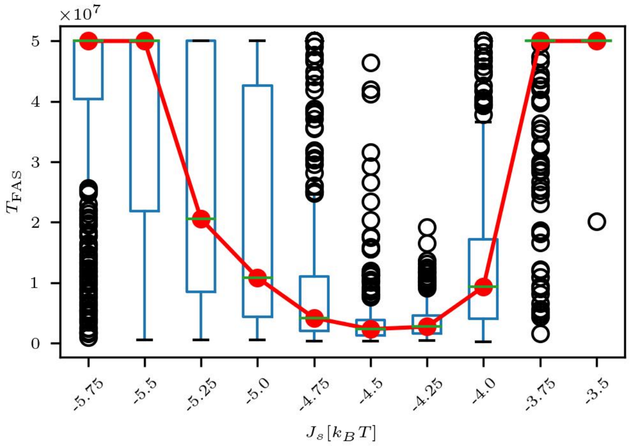

Figure S4: Boxplots of $T_{\rm FAS}$ across multiple J_s values for undirected VMMC simulation configuration with $\Delta\mu = -0.25$ $[k_BT]$.

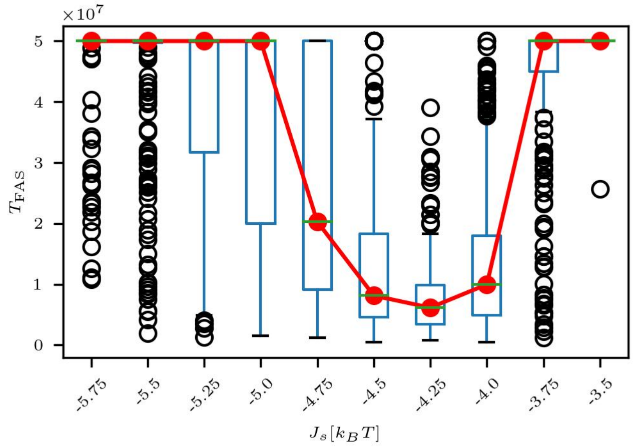

Figure S5: Boxplots of T_{FAS} across multiple J_s values for undirected SPMC simulation configuration with $\Delta\mu = -0.5$ $[k_BT]$.

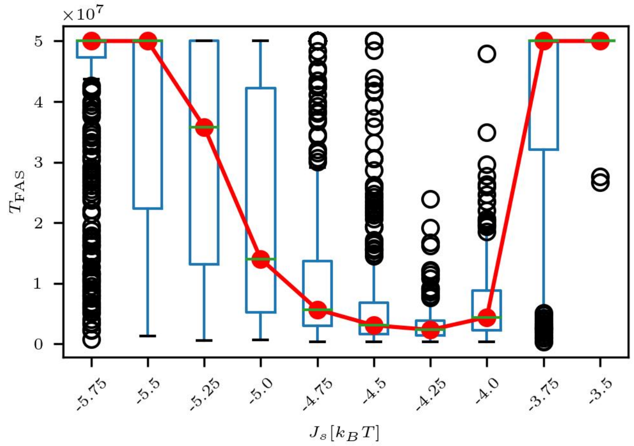

Figure S6: Boxplots of T_{FAS} across multiple J_s values for undirected VMMC simulation configuration with $\Delta\mu = -0.5$ $[k_BT]$.

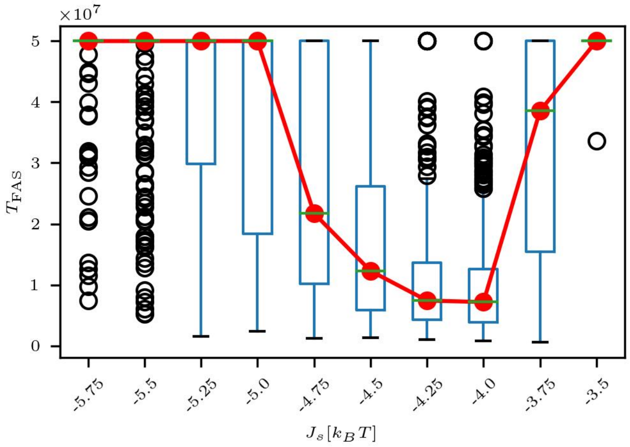

Figure S7: Boxplots of T_{FAS} across multiple J_s values for undirected SPMC simulation configuration with $\Delta\mu = -0.75$ $[k_B T]$.

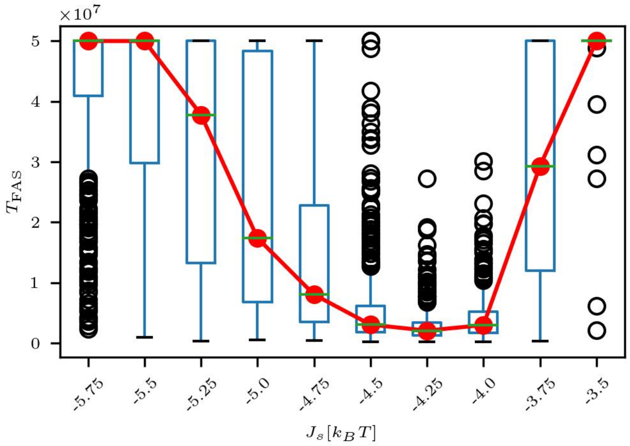

Figure S8: Boxplots of T_{FAS} across multiple J_s values for undirected VMMC simulation configuration with $\Delta\mu = -0.75$ $[k_B T]$.

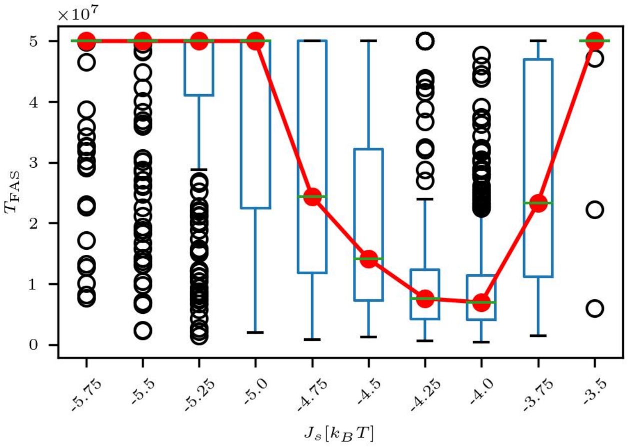

Figure S9: Boxplots of T_{FAS} across multiple J_s values for undirected SPMC simulation configuration with $\Delta\mu = -1$ $[k_BT]$.

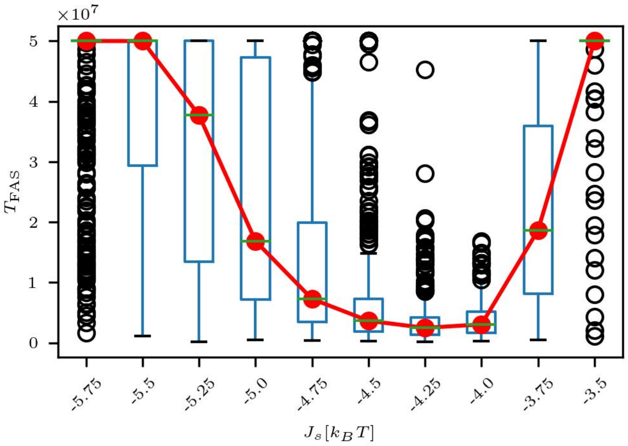

Figure S10: Boxplots of T_{FAS} across multiple J_s values for undirected VMMC simulation configuration with $\Delta\mu = -1$ $[k_BT]$.

S1.2 Assembly Pathways Heterogeneity Analysis

To characterize kinetic differences between simulation types, we employ a functional boxplot analysis.[2] Specifically, we first compare the one-dimensional assembly pathway variations of energy and maximum cluster size (MCS) time traces obtained from VMMC and SPMC simulations (Figs. S11 and S12, respectively; both under undirected interactions, abbreviated as UDI), measured in consecutive Monte Carlo (MC) sweeps. We then analyze kinetic differences between VMMC with directed interactions (DI) and VMMC with UDI by examining their $T_{\text{FAS}}(\Delta\mu)$ patterns (Fig. S13) and applying functional boxplot analysis to the corresponding trajectories (Figs. S14 and S15).

The functional boxplots were generated from ensembles of 15 simulated trajectories per simulation type at fixed drive values. Each drive value appears in the title of the corresponding subfigure, and the results for each drive were analyzed separately. For each time step in a trajectory ensemble, the median, 25%, and 75% percentiles of the observed quantity (energy or MCS) were calculated and plotted. The median trajectory appears as a solid line, and the interquartile range (IQR, i.e., the central 50% envelope) is shown as a shaded region. In this section, the IQR is considered a measure of uncertainty. Simulation results were truncated after 6000 MC sweeps, beyond which no significant dynamical changes were observed.

From Figs. S11 and S12, we observe across all drives that VMMC produces sharper and more consistent kinetic pathways than SPMC, as reflected in the narrower IQRs of VMMC trajectories. To quantify this difference, we computed the time-averaged IQR of the trajectories across drives for both methods. The results are summarized in Table S1 (energy, denoted as E) and Table S2 (MCS), where the left columns list SPMC results and the middle columns list VMMC results. For all drives and both observables, VMMC consistently exhibits smaller IQR values than SPMC, except at $|\Delta\mu| = 1.25$ $[k_B T]$, where their uncertainties are comparable.

To further probe kinetic differences, we interpret Fig. S12 trajectories within the first stable assembly seed-and-growth framework.[3] We find that the rate-limiting step of assembly

is similar for both simulation types: trajectories consistently begin to diverge once the MCS reaches $\sim$ 10 bonded particles. Since neither plot's IQR shows a tendency to return to smaller MCS values over time, we heuristically designate this assembly size as the stable assembly seed and the onset of the growth stage.

While stable assembly seed formation patterns are similar, the growth and stabilization phases differ markedly between SPMC and VMMC. For $\Delta\mu = -0.5$ $[k_BT]$, VMMC demonstrates steadier and faster growth toward larger cluster sizes than SPMC, as indicated by the quicker convergence of its median pathway to a plateau. After the plateau, SPMC shows substantially higher uncertainty than VMMC. For $\Delta\mu = -0.75$ $[k_BT]$, both methods exhibit a kinetic bottleneck, stalling near MCS $\sim$ 15. However, the bottleneck persists much longer under SPMC, with greater uncertainty, supporting the hypothesis that VMMC more efficiently escapes kinetic traps. At $\Delta\mu = -1.0$ $[k_BT]$ and -1.25 $[k_BT]$, VMMC again shows faster growth toward the target plateau and reduced uncertainty relative to SPMC.

This improved consistency and speed of VMMC assembly pathways is hypothesized to stem from its enhanced ability to maintain correct clusters through the multiple virtual moves attempted prior to executing the actual move,[4] as well as its capacity to efficiently disrupt incorrect kinetic traps via collective-mode dissociation.

Table S1: Average of the interquartile range values along the median energy trajectory (E) for all simulation types. IQR superscript indicates the interaction type, and the subscript indicates the simulation method.

$\lvert\Delta\mu\rvert$ $[k_BT]$	$IQR^{\mathrm{UDI}}_{\mathrm{SPMC}}(E)$	$IQR^{\mathrm{UDI}}_{\mathrm{VMMC}}(E)$	$IQR^{\mathrm{DI}}_{\mathrm{VMMC}}(E)$
0.5	70.12	51.53	14.71
0.75	34.51	22.39	12.84
1.0	30.11	17.26	10.64
1.25	23.6	24.47	7.65

Table S2: Average of the IQR values along the median Maximum Cluster Size (MCS) trajectory for all simulation types. IQR superscript indicates the interaction type, and the subscript indicates the simulation method.

$\|\Delta\mu\|$ $[k_BT]$	$IQR^{\text{UDI}}_{\text{SPMC}}(MCS)$	$IQR^{\text{UDI}}_{\text{VMMC}}(MCS)$	$IQR^{\text{DI}}_{\text{VMMC}}(MCS)$
0.5	10.02	6.52	1.9
0.75	4.58	2.57	1.65
1.0	3.27	1.74	1.44
1.25	2.77	2.63	1.02

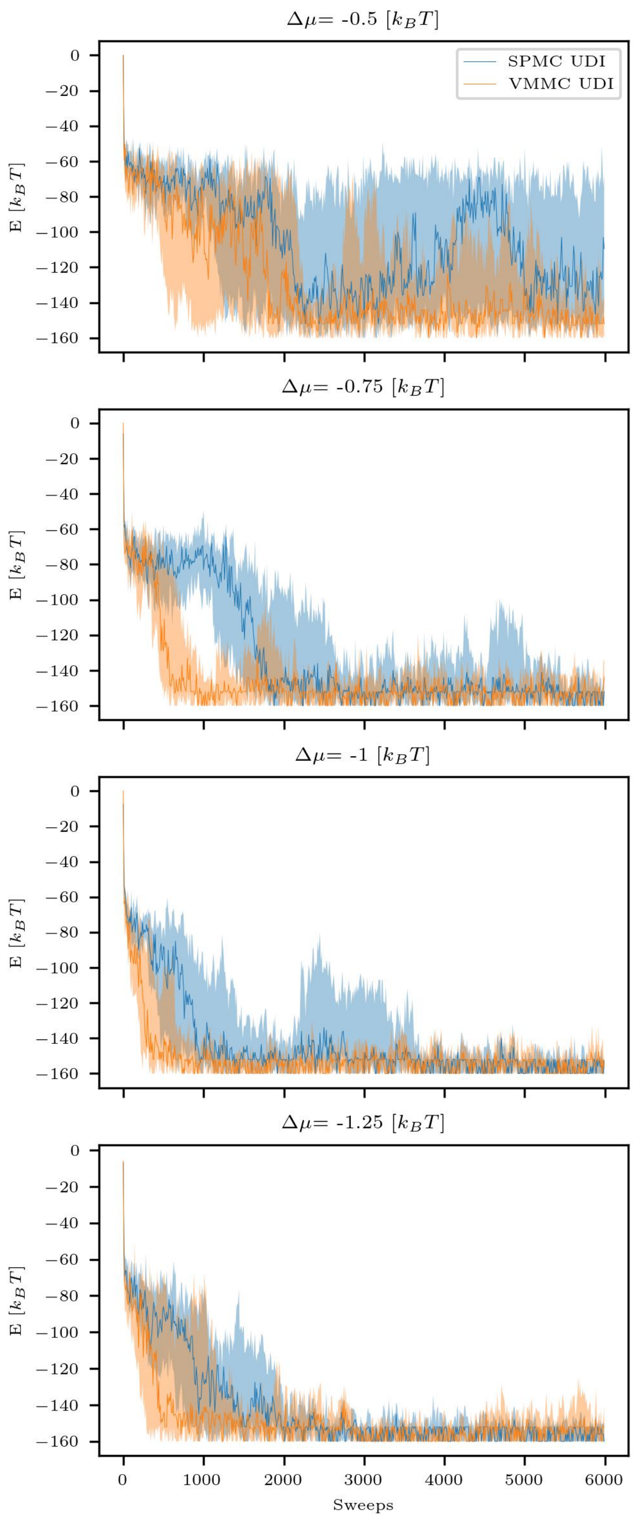

Figure S11: Energy trajectory uncertainty analysis for SPMC UDI and VMMC UDI configurations under different drive values, shown as energy versus MC step sweeps. The blue curves correspond to VMMC DI, and the orange curves to the VMMC UDI configuration. In all panels, the median trajectory is shown as a bold solid line, while shaded regions indicate the 25%–75% interquartile range of the simulated ensemble.

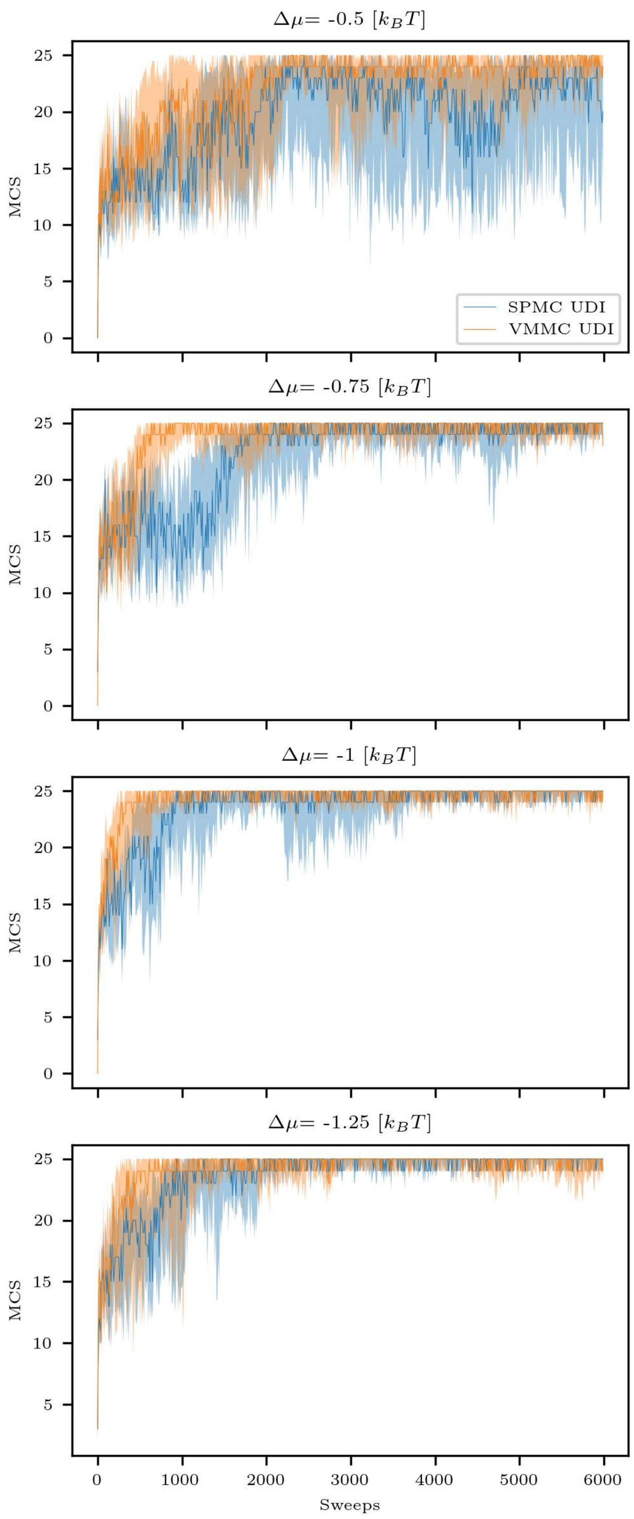

Figure S12: MCS trajectory uncertainty analysis for SPMC UDI and VMMC UDI configurations under different drive values, shown versus MC step sweeps. The blue curves correspond to VMMC DI, and the orange curves to the VMMC UDI configuration. In all panels, the median trajectory is shown as a bold solid line, while shaded regions indicate the 25%–75% interquartile range of the simulated ensemble.

We now compare DI and UDI kinetic pathways within VMMC. The first-assembly time T_{FAS} is plotted against the absolute drive magnitude $|\Delta\mu|$ on a logarithmic scale in Fig. S13. The results reveal an order-of-magnitude difference: UDI exhibits significantly longer assembly times. This disparity likely arises from the broader configurational space that UDI must explore before reaching the target structure, requiring more MC steps compared to the more constrained DI system. Despite this, both configurations show a consistent decrease in T_{FAS} with increasing $|\Delta\mu|$, indicating that nonequilibrium driving enhances assembly efficiency in a similar fashion across both cases. This trend suggests that the same underlying physical principles govern the acceleration of assembly in both directed and undirected interaction models.

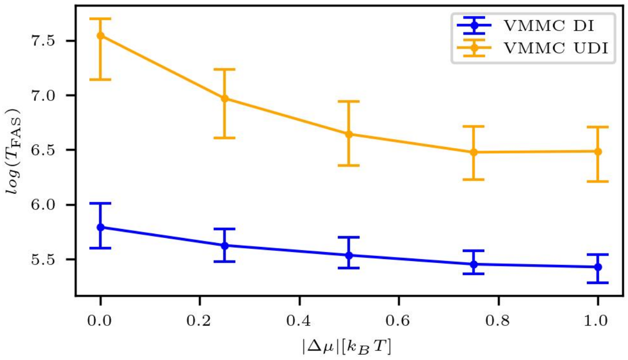

Figure S13: T_{FAS} comparison at $J_s = -4$ $[k_BT]$ across different drive values for VMMC UDI (blue) and VMMC DI (orange) configurations.

Finally, we inspect the DI and UDI kinetic pathways sweep-wise. As described in Table S1 of the main text, a DI sweep corresponds to $n_{\text{MC}} = 500$ MC steps, compared to $n_{\text{MC}} = 5000$ MC steps for UDI, consistent with the faster first-assembly times observed in Fig. S13. Sweep-wise comparison therefore highlights pathway heterogeneity beyond differences in overall assembly speed. The functional boxplots in Figs. S14 and S15 show that across drives, UDI generally exhibits larger IQRs than DI. In Fig. S15, the DI median pathway reveals a more consistent and less uncertain assembly progression, except at $\Delta\mu = -1$ $[k_BT]$. After plateauing, UDI exhibits significantly larger fluctuations in IQR than DI. To verify these visual observations, we computed time-averaged IQR values across

sweeps for both observables and all drives. Results are reported in Table S1 (energy) and Table S2 (MCS), where the middle column corresponds to UDI and the right column to DI. In all cases, DI exhibits significantly smaller IQRs than UDI, confirming that beyond faster assembly, DI produces more consistent and stable pathways.

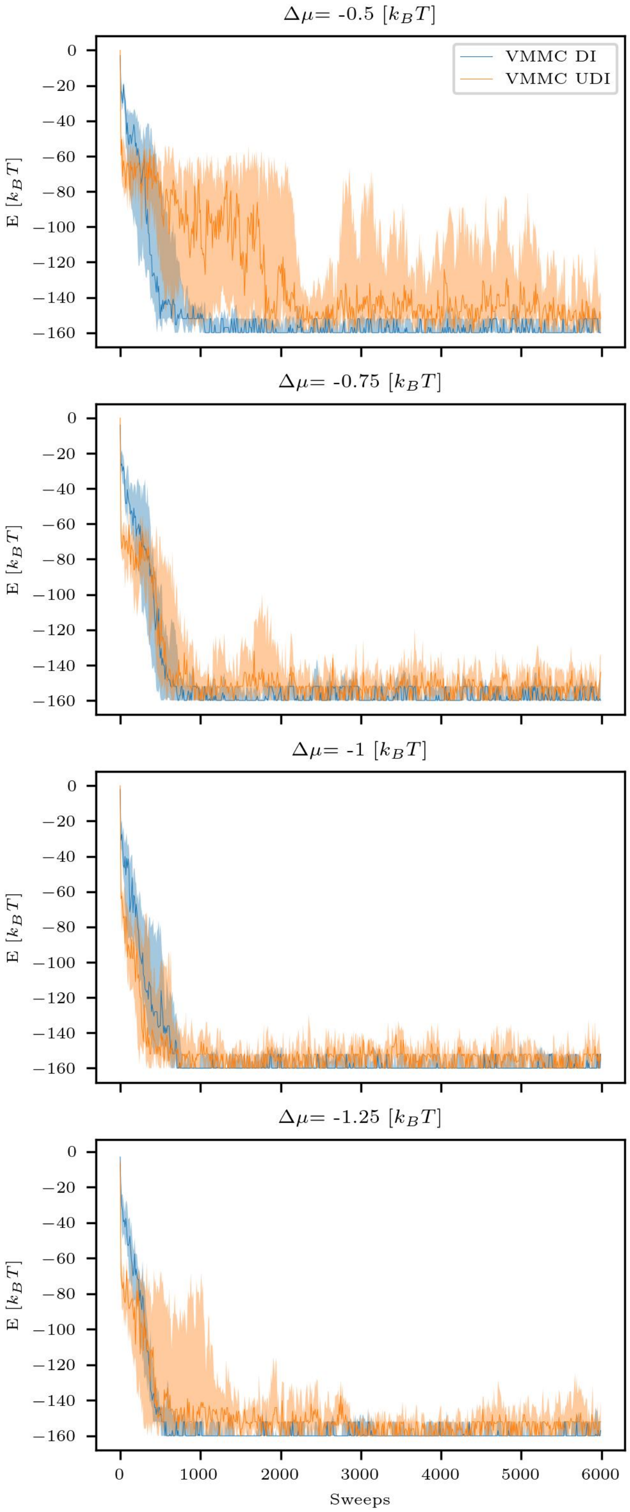

Figure S14: Energy trajectory uncertainty analysis for VMMC, comparing DI and UDI under different drive values, shown as energy versus MC step sweeps. The blue curves correspond to VMMC DI, and the orange curves to the VMMC UDI configuration. In all panels, the median trajectory is shown as a bold solid line, while shaded regions indicate the 25%–75% interquartile range of the simulated ensemble.

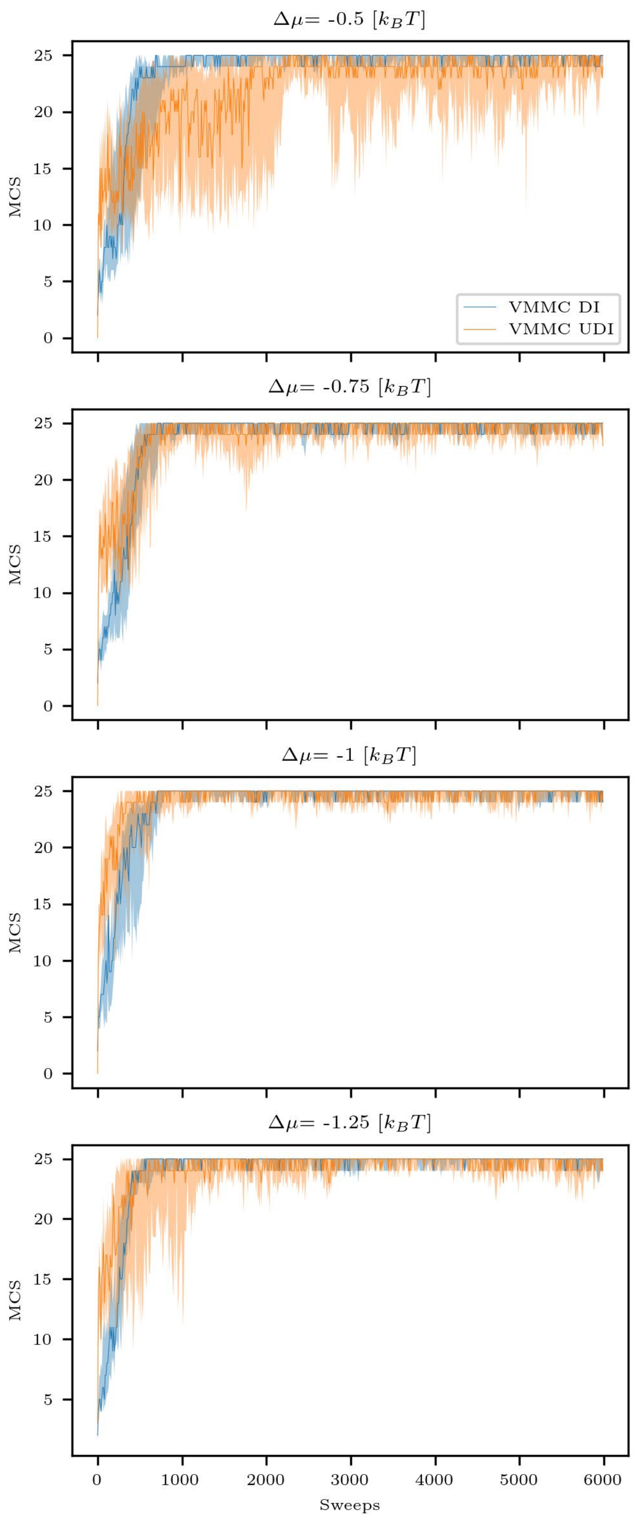

Figure S15: MCS trajectory uncertainty analysis for VMMC, comparing DI and UDI under different drive values, shown versus MC step sweeps. The blue curves correspond to VMMC DI, and the orange curves to the VMMC UDI configuration. In all panels, the median trajectory is shown as a bold solid line, while shaded regions indicate the 25%–75% interquartile range of the simulated ensemble.

References

(1) Faran, M.; Bisker, G. Nonequilibrium self-assembly time forecasting by the stochastic landscape method. *J. Phy. Chem. B* **2023**, *127*, 6113–6124.

(2) Sun, Y.; Genton, M. G. Functional boxplots. *J. Comp. Graph. Stat.* **2011**, *20*, 316–334.

(3) Kashchiev, D. On the relation between nucleation work, nucleus size, and nucleation rate. *J. Chem. Phys.* **1982**, *76*, 5098–5102.

(4) Whitelam, S.; Geissler, P. L. Avoiding unphysical kinetic traps in Monte Carlo simulations of strongly attractive particles. *J. Chem. Phys.* **2007**, *127*, 154101.
